# Crosslinker-free collagen gelation for corneal regeneration

**DOI:** 10.1038/s41598-022-13146-9

**Published:** 2022-06-01

**Authors:** Mohammad Mirazul Islam, Alexandru Chivu, Dina B. AbuSamra, Amrita Saha, Sumit Chowdhuri, Bapan Pramanik, Claes H. Dohlman, Debapratim Das, Pablo Argüeso, Jaya Rajaiya, Hirak K. Patra, James Chodosh

**Affiliations:** 1 Department of Ophthalmology, Massachusetts Eye and Ear and Schepens Eye Research Institute, Harvard Medical School, Boston, MA 02114 USA; 2 Department of Surgical Biotechnology, University College London, London, NW3 2PF UK; 3 Department of Chemistry, Indian Institute of Technology Guwahati, Guwahati, Assam 781039 India; 4 Department of Chemistry, Ben Gurion University of the Negev, Be’er Sheva, Israel

**Keywords:** Corneal diseases, Regenerative medicine, Tissue engineering, Biomaterials, Biomaterials - cells, Biomedical materials, Implants, Tissues, Gels and hydrogels

## Abstract

Development of an artificial cornea can potentially fulfil the demand of donor corneas for transplantation as the number of donors is far less than needed to treat corneal blindness. Collagen-based artificial corneas stand out as a regenerative option, having promising clinical outcomes. Collagen crosslinked with chemical crosslinkers which modify the parent functional groups of collagen. However, crosslinkers are usually cytotoxic, so crosslinkers need to be removed from implants completely before application in humans. In addition, crosslinked products are mechanically weak and susceptible to enzymatic degradation. We developed a crosslinker free supramolecular gelation strategy using pyrene conjugated dipeptide amphiphile (PyKC) consisting of lysine and cysteine; in which collagen molecules are intertwined inside the PyKC network without any functional group modification of the collagen. The newly developed collagen implants (Coll-PyKC) are optically transparent and can effectively block UV light, are mechanically and enzymatically stable, and can be sutured. The Coll-PyKC implants support the growth and function of all corneal cells, trigger anti-inflammatory differentiation while suppressing the pro-inflammatory differentiation of human monocytes. Coll-PyKC implants can restrict human adenovirus propagation. Therefore, this crosslinker-free strategy can be used for the repair, healing, and regeneration of the cornea, and potentially other damaged organs of the body.

## Introduction

Organ transplantation not only saves lives but also improves the quality of life for recipients. However, the demand for solid organs for transplantation is many folds higher than the availability. In the year 2018 (pre-pandemic condition), 140,964 organ transplantations were carried out worldwide^1^, however the recent data suggests that the number of transplantations fulfilled only 10% of the real need. Organ donation and transplantation became more difficult in the period of COVID-19^2^. Recent advancements in biomaterials development hold tremendous potential to fill the gap between the supply and demand of donated organs by developing tissue-engineered replacements. For engineered organ development, the key challenge is appropriate biomaterial selection and suitable assembly through crosslinking to form apposite scaffolds for the regeneration of the target organ. Chemical crosslinkers can improve the physicochemical properties of artificial biomaterials and decellularized organs^3^. However, the choice of crosslinkers is highly crucial for building and optimizing the properties of the scaffold, as most of these chemicals demonstrate negative effects on the cellular compatibility of the engineered tissue. This is because crosslinkers act on and modify existing functional groups such as free carboxylic acid (-COOH), primary amine (-NH_2_) within biomaterials, therefore altering the chemical identity and functional group content of the original/parent compound.

Over the past decades, the development of an artificial cornea has emerged as a prime research interest. It is estimated that about 285 million people in the world live with visual impairment, and 39 million of them are blind^4^. Corneal disease is one of the foremost causes of visual impairment^5^. Replacement of a diseased cornea with a donor cornea through transplantation is the most practiced clinical treatment. However, donor corneas are in short supply worldwide, resulting in 10 million untreated patients with 1.5 million additional every year^6^. It has been determined that we currently have only one donor cornea for every 70 persons in need^7^. Additionally, corneal allotransplantation is contraindicated in autoimmune disorders and after severe chemical injury^8^. Donor-derived infection is, furthermore, a significant complication^9^.

Many attempts have been made to find alternatives to corneal allografts, with limited success, but these efforts have contributed to increase our knowledge for the development of artificial corneas. In this context, synthetic polymer-based materials are mechanically robust but lack of cellular biocompatibility, that unlike natural polymer-based biomaterials^10,11^, limit their application. Among the natural polymers used for tissue engineering, collagen-based artificial corneas (CACs) have been extensively studied as a potential alternative for donor corneas for transplantation, as the human cornea is mainly made up of collagen^12^. CACs have been transplanted in humans and post-trial clinical results showed the capacity of the CACs placed as lamellar, partial thickness grafts to restore vision after transplantation in relatively non-severe corneal disease^13^. At 4 years follow-up, the corneas were well integrated into the host tissue. Interpenetrating polymer-based CACs have also been evaluated in a human pilot study^14^, and in a clinical trial^15^ and showed the capacity to stabilize corneas and improve vision in aggressive disease condition. Although CACs are highly biocompatible, their mechanical properties do not permit suturing of the implant and require overlying sutures for transplantation^13^. Enzymatic susceptibility of CACs is another weakness concerning their implantation in severely diseased eyes. Hence, an implant is needed that is enzymatically stable and permits the peripheral placement of interrupted or continuous sutures, therefore avoiding crossing the central cornea with overlying sutures with the attendant scarring and altered topography that each reduce visual acuity^16^.

Furthermore, corneal perforations remain an urgent, vision-threatening problem usually requiring immediate sealing of the cornea. The most applied clinical practice involves temporarily sealing the cornea with cyanoacrylate glue in order to maintain globe integrity and save the eye, as initiated in the late 1960s^17,18^. However, cyanoacrylate has low biocompatibility^17^ and causes inflammation^19^. Incomplete polymerization leaves behind toxic cyanoacrylate monomers that can undergo hydrolysis to release potentially toxic compounds such as formaldehyde and alkyl cyanoacrylate^20^. These can induce further corneal injury through scarring and neovascularization^20^. Many patients treated with cyanoacrylate eventually require full corneal transplantation. Considering these limitations, patient care would benefit from a nontoxic sealant for perforated corneas.

In this present work, we describe the development of a crosslinker-free collagen scaffold for corneal regeneration. We have previously reported a self-assembly process of a small peptide (PyKC) based gelation strategy to form a water-insoluble supramolecular hydrogel that remained insoluble for more than a year in water and various other solutions^21^. The unique confinement property of this peptide hydrogel can be used to efficiently trap and protect protein molecules from external denaturing agents for a prolonged period^21^. The current study used 1 and 2% PyKC as an assembling units to stitch together the extracellular matrix (ECM) protein collagen (type I) to form CACs. This implant can be transplanted as a substitute for a human donor cornea in patients who would otherwise receive a penetrating keratoplasty. The pyrene modified dipeptide underwent a self-assembly process through hydrophobic interactions between the pyrene rings, as well as via the formation of disulphide bridge (–S–S–) between cysteine residues, which eventually entrapped collagen to form the hydrogel without modifying the collagen molecules as such. This new formulation (Coll_x_-PyKC_y,_ where x and y are wt% of respective component) can be used to develop a stand-alone artificial cornea or directly on the eye to work as a sealant to repair corneal perforations. In principle, the proposed strategy can further be used to assemble and embed proteins/peptides irrespective of their size, enzymes, and small molecule as a delivery platform for ophthalmic or other applications. The gelation process is not instantaneous; therefore, it allows for adequate homogeneous mixing time. The formulation components are nontoxic, so the homogeneous mixture of PyKC and collagen can be directly applied on organs/wound sites and over time will form a gel. Our Coll_x_-PyKC_y,_ hydrogels are stable and resistant to collagenase degradation and exhibit dose-dependent protection with increasing PyKC units (y).

## Materials and method

Type I porcine atelocollagen was purchased from Nippon Meat Packers Inc. (Tokyo, Japan). PyKC was synthesized according to a previously published report^21^. All reagents were of analytical grade and used as received. All chemicals were procured from Sigma-Aldrich (St. Louis, MO) if not mentioned otherwise.

### Fabrication of collagen hydrogel

Control collagen hydrogels were made following a previously published protocol with modifications^22^. In brief, starting solution containing 10% or 15% (w/w) collagen and 1-ethyl-3-(3-dimethylaminopropyl) carbodiimide hydrochloride (EDC)-to-collagen primary amine group ratio was kept at 0.7:1.0 molar equivalent. 0.5 mL aliquots of collagen solution was loaded into a syringe mixing system^23^. The syringe mixing system was pre-filled with 0.625 M 2-morpholinoethanesulfonic acid monohydrate (MES) buffer. Excess MES solution was added to the collagen to keep the dilution factor similar for all hydrogels. The collagen solution was then adjusted to pH 5 ± 0.5 with microliter quantities of 2 N aqueous NaOH, followed by thorough mixing. Calculated volumes of aqueous solutions of EDC and its co-reagent N-hydroxysuccinimide (NHS; both at 10% [w/v]) were added to give a 2:1 molar ratio of EDC to NHS and mixed with the collagen solution. The final homogeneous solution was immediately dispensed between two glass slides 500 μm apart. After 24 h, the hydrogels were washed three times with 1 × Phosphate-buffered saline (PBS). Before performing biocompatibility studies, the hydrogels were treated with 3X antibiotic solution consisting of 300 unit/mL penicillin and 300 µg/mL streptomycin as previously described^16^.

For PyKC collagen hydrogels, the above syringe mixing system was used, but the mixing system was prefilled with 20 mM Tris–HCl buffer (pH 8.0). The collagen solution was adjusted to pH 9 ± 0.5 with microliter quantities of 2 N aqueous NaOH, followed by thorough mixing. 100 μL PyKC solution for PyKC_1_ (1% PyKC containing hydrogel) or 150 μL of PyKC solution for PyKC_2_ (2% PyKC containing hydrogels) was added to the respective collagen solutions in 20 mM Tris–HCl buffer. The homogeneous collagen mix was cast between glass slides spaced 500 µm apart, and follow-up processing was performed as with the control hydrogel. (Hydrogels of 10% and 15% collagen (as controls), 10% collagen and 1% PyKC, 10% collagen and 2% PyKC, 15% collagen and 1% PyKC, and 15% collagen and 2% PyKC and, are referred to as Coll_10_, Coll_15_, Coll_10_-PyKC_1_, Coll_10_-PyKC_2_, Coll_15_-PyKC_1_ and Coll_15_-PyKC_2_, respectively).

### Optical transmission

The optical transmission of each hydrogel was analyzed by a UV-Vis spectrometer (Molecular Devices SpectraMax 384 Plus Microplate Reader, Molecular Devices; San Jose, CA). Six mm diameter trephined round hydrogels (N = 3) were placed in individual wells of a 96-well quartz microplate. The transmittance of light through the hydrogels was recorded at wavelengths ranging from 200 to 800 nm at 1 nm wavelength increments. The transmittance of the samples was corrected with blank media (H_2_O) and the mean transmittance (%) for each group was calculated and plotted against individual wavelength.

### Water content measurement

The water content of hydrogels (N = 4) was determined to ensure uniformity using a previously published protocol^24^ with minor modifications. In brief, hydrated hydrogels were removed from PBS, the surface was gently blotted and then the samples immediately weighed on a microbalance to record the wet weight (W_0_) of the hydrogels. Dry weights of the same hydrogels were measured after drying the samples at 50 °C until a constant weight was achieved (W).

The equilibrated water content of the hydrogels (W_t_) was calculated according to the following equation: W_t_ = (W_0_-W)/W_0_ × 100%.

### Suture pull-out study

A special setup was used to evaluate the suture pull out resistance^25^. Six mm diameter round hydrogels (N = 3) were placed on a stage and a suture (Nylon suture size 9–0) was placed in the middle of the hydrogel and connected to the bottom part of the machine. The other end of the suture was also attached to the bottom of the machine (Mark-10 ESM 303, Copiague, NY) through the cut located in the middle of the stage. To measure the resistance to suture cutting through the hydrogels, the stage was pulled up and the energy required to cut the hydrogel entirely and remove the suture from each hydrogel was measured.

### Fourier-transform infrared spectroscopy (FT-IR)

FT-IR was performed on a Jasco attenuated total reflectance FT-IR 4200 spectrometer (Jasco ATR FT/IR-4200, Japan), averaging 30 scans between 4000 and 600 cm^-1^, at a resolution of 2 cm^-1^. The measurements were performed on samples in hydrated form, as well as after drying in a vacuum desiccator for 24 h (N = 3).

### Contact angle measurement

Contact angle measurements were performed to measure the surface hydrophilicity of different hydrogel samples (N = 3). A drop of 3 μL dH_2_O was deposited onto each hydrogel by a micro-syringe and images were taken with a Dino-light digital microscope (AnMo Electronics Corporation, Hsinchu, Taiwan). The contact angle was measured with ImageJ (U.S. National Institutes of Health, Bethesda, Maryland, USA).

### Differential scanning calorimetry (DSC)

The thermal properties of the hydrogels (N = 4) were tested using a DSC 1 STAR System (Mettler-Toledo, Columbus, OH). Heating scans were recorded within the range of 10–80 °C at a scan rate of 1 °C/min. Pre-weighed samples of the PBS-equilibrated hydrogels (weights ranging from 8 to 17 mg) were surface-dried with filter paper and hermetically sealed in an aluminium pan to prevent water evaporation during the study. A resulting heat flux versus temperature curve was then used to calculate the denaturing temperature (T_m_). T_max_ of the endothermic peak gives the denaturing temperature.

### In vitro biocompatibility

Because the proposed crosslinker-free biomaterials were intended to be used as artificial corneas, human corneal cells were used to evaluate hydrogel biocompatibility. The human cornea is composed of three primary cellular layers, an outermost epithelium, a middle stroma containing fibroblast-like keratocytes and an innermost, single layer of endothelial cells^26^. Each of these corneal cell populations is important for the function of the cornea, and individual cell types can behave differently in response to specific properties of an implanted biomaterial. Hence, we have evaluated the biocompatibility of all three cell types with the respective collagen hydrogels. Cells grown on collagen hydrogel crosslinked with EDC/NHS and on Tissue culture plate (TCP) were used as controls.

#### Human corneal epithelial cells (HCEC)

The biocompatibility of the hydrogels was tested using SV40-immortalized HCEC, as previously reported^24^, and kindly provided by Professor May Griffith. The hydrogels were cut into 6 mm diameter segments and 10,000 HCEC were seeded on top of each for culture with DMEM/Ham's F-12 medium (Corning, Manassas, VA, USA) supplemented with 10% Newborn Calf Serum (NCS) (HyClone, Logan, Utah, USA), 10 ng/mL epidermal growth factor (EGF) (Gibco, California, USA), and 1% penicillin/streptomycin (Gibco, NY, USA), at 37 °C and 5% CO_2_^27^. The cell culture medium was changed every other day. Alamar Blue staining was performed on day 1, day 4 and day 6 after cell seeding. At each time point, 4 μL resazurin sodium salt solution was added to the cell culture medium to give a final concentration of resazurin sodium salt at 0.004% w/v, and incubated for 3 h. Afterward, the cell-incubated resazurin sodium salt solution was diluted with fresh culture medium in a new 96 well plate and read on a BioTek plate reader (Synergy 2, BioTek Instruments; Winooski, VT) at 530/25 nm for excitation and 590/35 nm for emission. Relative fluorescence units were obtained by multiplying the measured values with the dilution factors. A cytotoxicity study was performed on the day 1, day 4 and day 6 through live/dead staining (Life Technologies Corporation, Oregon, USA), where cells were double-stained by calceinacetoxymethyl (Calcein AM) and ethidium homodimer-1 (EthD-1). Live cells and dead cells stained as green and red, respectively. Images were captured by using a fluorescence microscope (Zeiss Axio Observer Z1, Carl Zeiss Microimaging GmbH, Jena, Germany).

For histological evaluation, the hydrogels were fixed in formaldehyde, dehydrated in increasing concentrations of ethanol (70, 96, 100%) for 30 min at each concentration, immersed twice in xylene for 30 min, and then kept in liquid paraffin for 30 min. Paraffin-embedded sections were cut to 6 μm thickness with a microtome and stained for histology with hematoxylin for 2 min and eosin for 1 min (H&E, Hematoxylin Stain, Fisher Chemical, NJ, USA; Eosin Y, Fisher Chemical).

#### Human corneal fibroblasts (HCF)

Immortalized human corneal fibroblasts (HCF), derived from keratocytes and kindly donated by Professor James V Jester, were used for evaluating the biomaterials. 5000 HCF were cultured on the top of each hydrogel and cultured with DMEM/Ham's F-12 media supplemented with 10% fetal bovine serum (FBS) (Life Technologies) for six days at 37 °C in 5% CO_2_. Alamar Blue assay was performed at days 1, 4, and 6 after cell seeding and live/dead staining was performed on the day 1, 4, and 6.

#### Human corneal endothelial cells (CEC)

Telomerase immortalized human corneal endothelial cells (CEC) were kindly provided by Professor Ula Jurkunas. Cells were cultured in Opti-MEM I with Glutamax-I media (Life Technologies) supplemented with 8% (v/v) FBS, 5 ng/mL EGF (EMD Millipore Corporation, Temecula, CA), 0.2 mg/mL calcium chloride (Fisher Scientific Company, Fair Lawn, NJ), 0.8 mg/mL chondroitin sulfate-A (Sigma-Aldrich, St. Louis, MO), 0.25 mg/mL Gentamycin (Life Technologies), 1% (v/v) Antibiotic–Antimycotic solution (Life Technologies), and 0.1 mg/mL bovine pituitary extract (Alfa Aesar, Ward Hill, MA) for 6 days. 5000 CEC were seeded on the top of each hydrogel and incubated at 37 °C in 5% CO_2_. TCP was coated with cell attachment enhancer coating (FNC Coating Mix, Athena Environmental Sciences, Inc, Baltimore, MD) before seeding the cells. Alamar Blue assay was performed at day 1, 4, and 6 after cell seeding, and live/dead staining was performed on the day 1, 4, and 6.

### Immunofluorescence microscopy

The expression of cytokeratin 3 + 12 and the mucin MUC16 by HCEC after culture on different hydrogels was determined by immunofluorescence microscopy, using paraffin embedded tissue sections. The same paraffin embedding protocol was used as described above. Staining was performed according to a previously published protocol^28^. Paraffin was removed from the tissue sections using xylene, and the samples were rehydrated in water through a graded series of alcohols (100, 96, 70, 50%, and water). For antigen retrieval, tissue sections were incubated with 10 mM Sodium citrate buffer, 0.05% Tween 20 (pH 6.0) at 60 °C for overnight. The sections were washed with Tris-buffered saline (TBS) plus 0.025% Triton X-100 followed by blocking any unspecific binding sites using TBS supplemented with 10% FBS and 1% bovine serum albumin (BSA). The sections were then incubated with the primary antibodies overnight at 4 °C in humidifying condition. Corneal epithelial cell specific mouse cytokeratin (anti-cytokeratin 3 + 12, clone AE5, ab68260, Abcam) and mouse monoclonal antibody against corneal mucin (anti-MUC16, clone X75; ab10029, Abcam) were used separately at dilution 1:50 and 1:300, respectively. Incubation with secondary antibodies was carried out for 1 h at room temperature. FITC-conjugated anti-mouse antibody (ab6785, dilution 1:100; Abcam) was used as secondary antibody. Finally, slides were mounted with EthD-1 and finally with VectaShield mounting medium containing DAPI (Vector Laboratories, Inc., CA, USA), and examined by an inverted fluorescent microscope (Zeiss Axio Observer Z1) with a 40 × objective.

To evaluate the expression of ALDH3A1 and alpha smooth muscle actin (α-SMA) marker by primary (p^1^) human corneal fibroblasts (pHCF) cultured on different hydrogels, we used standard immunocytochemistry assay. pHCF was cultured from deceased donor corneas deemed unsuitable for human transplantation. Briefly, pHCF (10,000 cells per well) were seeded on the hydrogels and cultured for three days, then fixed in paraformaldehyde (4%). For permeabilization and blocking, hydrogels were incubated in PBS containing Triton X-100 (0.25%) for 10 min and PBST (PBS with 0.05% Tween-20) containing 5% FBS for 1 h, respectively. Each hydrogel was then incubated with the mouse monoclonal antibody against ALDH3A1 (clone 1B6; dilution 1:100, GTX84889, GenTex, Irvine, CA); and with mouse monoclonal antibodies against α-SMA (clone 1A4; dilution 1:200, ab7817, Abcam) for overnight at 4 °C. Then, each hydrogel was incubated with FITC-conjugated anti-mouse antibody (dilution 1:1000, ab6785, Abcam) as a secondary anti-body for 1 h at room temperature. Finally, the slides were mounted in VectaShield mounting media containing DAPI and imaged by an inverted fluorescent microscope (Axio Observer Z1, Zeiss) with a 10 × objective.

Standard immunofluorescence assay was also used to evaluate the expression of ZO-1 marker by CEC cultured on the hydrogels^29^. Briefly, CEC (10,000 cells per well) were seeded and on day three, the hydrogels were washed and fixed with paraformaldehyde (4%). Permeabilization and blocking were done as mentioned above. The hydrogels were then incubated with the rabbit polyclonal antibody against ZO-1 (ZO-1 Polyclonal Antibody, dilution: 1:100, 402,200, ThermoFisher Scientific) as a primary antibody overnight at 4 °C in humidifying condition^29^. Afterward, the specimens were incubated with FITC-conjugated anti-Rabbit antibody (dilution: 1:600, ab6717, Abcam) as a secondary antibody for 1 h at room temperature. Samples were then stained with EthD-1. Images were taken by a fluorescent microscope (Axio Observer Z1, Zeiss) with 100 × objective.

### Protection against adenovirus type 37

Real-Time PCR: For viral infection, HCEC were seeded at 10,000 cells/well on 96 well plates on different hydrogels (hydrogel-cells) along with only cells or hydrogel-no cells controls. At 80–90% confluency, cells were washed and were infected with human adenovirus species D type 37 (HAdV-D37, GenBank Acc No. DQ900900) at a multiplicity of infection (MOI) of 1 in keratinocyte serum-free medium (KSFM). After 1 h of adsorption at 37 °C, each well was washed and replenished with media mentioned in Sect. 2.10.1. Mock infected control, and virus-only control were kept alongside. Infected hydrogel-cell constructs, hydrogel-no cell, mock cell control, and virus-only controls were harvested at 24 h post infection (hpi). According to manufacturer’s protocol, DNA was isolated using DNeasy Blood & Tissue Kits (Qiagen, USA). To quantify viral DNA replication, the HAdV-D37 E1A gene was amplified after 24 hpi. For PCR, 5 µg of DNA was used as a template. Primer pairs for EIA (sense 5’-CAGTCCATGCCATCACTGCCACCCA-3’; antisense 5’-CAGGGATGACCTTGCCCACAGCCTT-3’) were used for gene amplification. Real-time PCR (RT-PCR) was performed using Fast SYBR Green master mix (Applied Biosystems, Foster City, CA) with the cycling parameters: stage 1, 50 °C for 2 min; stage 2, 95 °C for 10 min; stage 3 (40 cycles) 95 °C for 15 s, 60 °C for 1 min, and stage 4, 95 °C for 15 s, 60 °C for 15 s and 95˚C for 15 s. The amplified product was verified by melting curve analysis using QuantStudio™ Design & Analysis software v1.5.0 (Applied Biosystems, Thermo Fisher Scientific, USA). A no-template control and an endogenous control (GAPDH) were measured, and the expression levels were calculated by the 2^-ddCt method and compared with the mock and infected controls.

Immunofluorescence assay: Cell culture conditions, and virus infection were the same as described above. At 48 hpi, HCEC cultured on different hydrogels were fixed and ICC was performed. Cells were incubated with anti-adenovirus type 5 antibody (rabbit polyclonal to adenovirus type 5, ab6982, dilution 1:800, Abcam) for overnight, followed by immunostaining with FITC conjugated anti-rabbit antibody (ab6717, dilution 1:1000, Abcam) for 1 h at room temperature. Pictures were taken with a 40 × objective. EthD-1 was added to the hydrogel before adding the DAPI containing mounting media to double stain the cell nuclei.

### Hydrogels and their influence on adaptive immunity

Human monocyte THP-1 cells were used to determine the effect of the hydrogels on adaptive immunity. THP-1 cells were cultured on the hydrogels in RPMI (Gibco) media supplemented with 10% FBS, 1% penicillin/streptomycin and 50 μM β-mercaptoethanol (Gibco) (Standard media) and cultured for 7 days at 37 °C in 5% CO_2_. To induced differentiation of the monocytes to antigen presenting dendritic cells (APCs), the standard media was supplemented with 100 ng/mL rhIL-4 (R&D System, Minneapolis, USA) and 100 ng/mL rhGM-CSF (R&D System). THP-1 cells cultured on TCP with or without differentiation ingredients were used as negative controls. For a positive control, another set was performed on TCP where standard media was supplemented with differentiation ingredients together with lipopolysaccharide (1 × LPS, Invitrogen, Carlsbad, CA, USA). Morphological changes of the cells were evaluated by 20 × phase-contrast images taken at days 1 and 7 with Nikon Eclipse (TS100) microscope. The influence of hydrogels on monocyte differentiation with or without differentiation ingredients was evaluated by labeling the cells after culture for 7 days with direct-conjugate antibodies against CD86 (pro-inflammatory activated APCs), and CD206 (inactivated APCs marker) (Table 1). Data was acquired using a BD LSR II and the flow cytometry results were analyzed using FlowJo™ v10.6.1 Software (BD Life Sciences)^30^.

**Table 1:** Antibodies for flow cytometry.

Target	Antibody	Supplier	Dilution factor
CD86	APC Mouse Anti-Human CD86, Clone 2331 (FUN-1)	BD Bioscience, MD, USA	1/20
Isotype Control for CD86	APC Mouse IgG1, κ, Clone MOPC-21	BD Bioscience	1/20
CD206	PE Mouse Anti-Human CD206, Clone 19.2	BD Bioscience	1/20
Isotype Control for CD206	PE Mouse IgG1, κ, Clone MOPC-21	BD Bioscience	1/20

### Statistical analysis

Data are shown as averages ± standard deviations, and one-way ANOVA with Tukey post hoc test performed to compare groups. A value of *p* < 0.05 was considered statistically significant. n.s., *, **, *** and **** represent *p* > 0.05, *p* < 0.05, *p* < 0.01, *p* < 0.001 and *p* < 0.0001, respectively. GraphPad Prism Software (GraphPad Software, CA, USA) was used to analyze the data.

## Results

**Figure 1:**
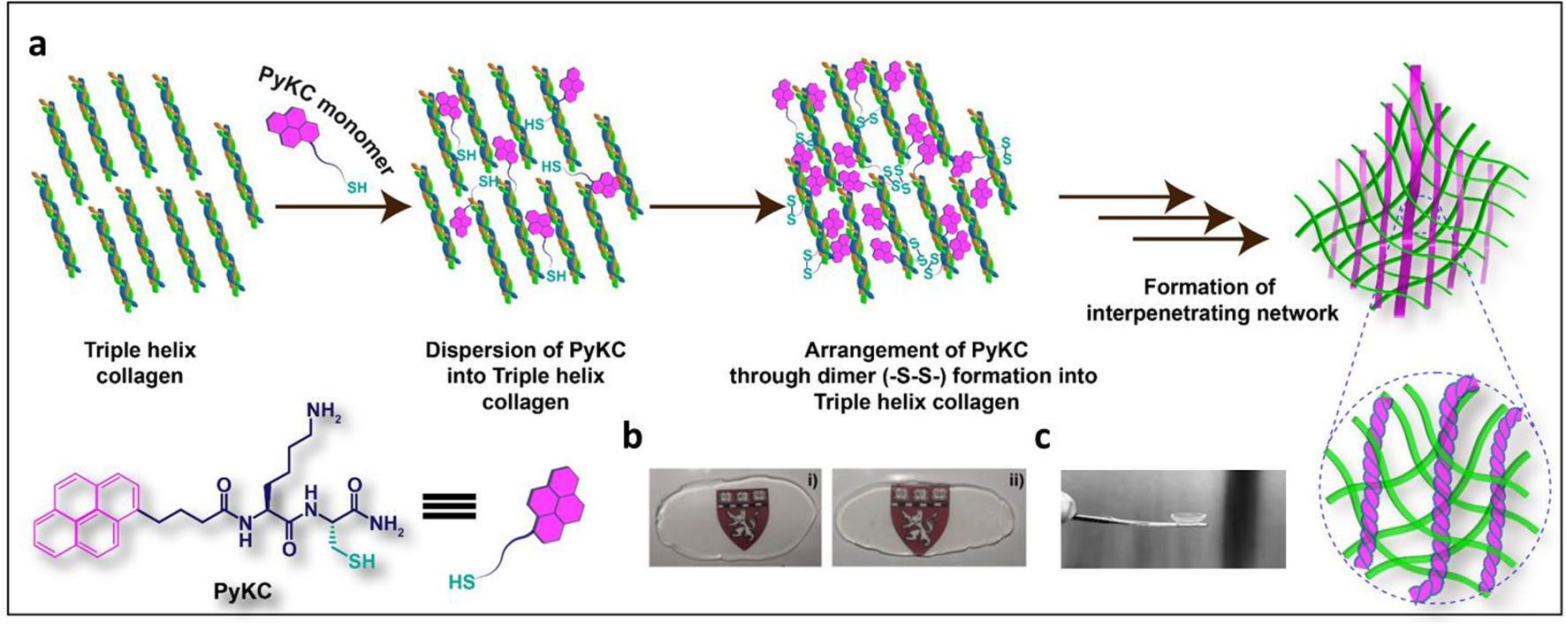
(**a**) Schematic representation of the crosslinker free Coll-PyKC hydrogel generated by forming an interpenetrating network. (**b**) Transparency of the hydrogels as illustrated by photography of Coll-PyKC hydrogels from Coll_10_-PyKC_1_ (i), Coll_10_-PyKC_2_ (ii) on Harvard logo. (**c**) Photograph of cornea-shaped hydrogel prepared from Coll_10_-PyKC_2_ hydrogel. In Coll_x_-PyKC_y,_ weight percentage of collagen and PyKC are denoted as (x) and (y), respectively.

Hydrogels generated from PyKC and collagen were transparent and could be cast as plain sheets or curvature similar to a human cornea (Fig. 1).

### Optical transmission

**Figure 2:**
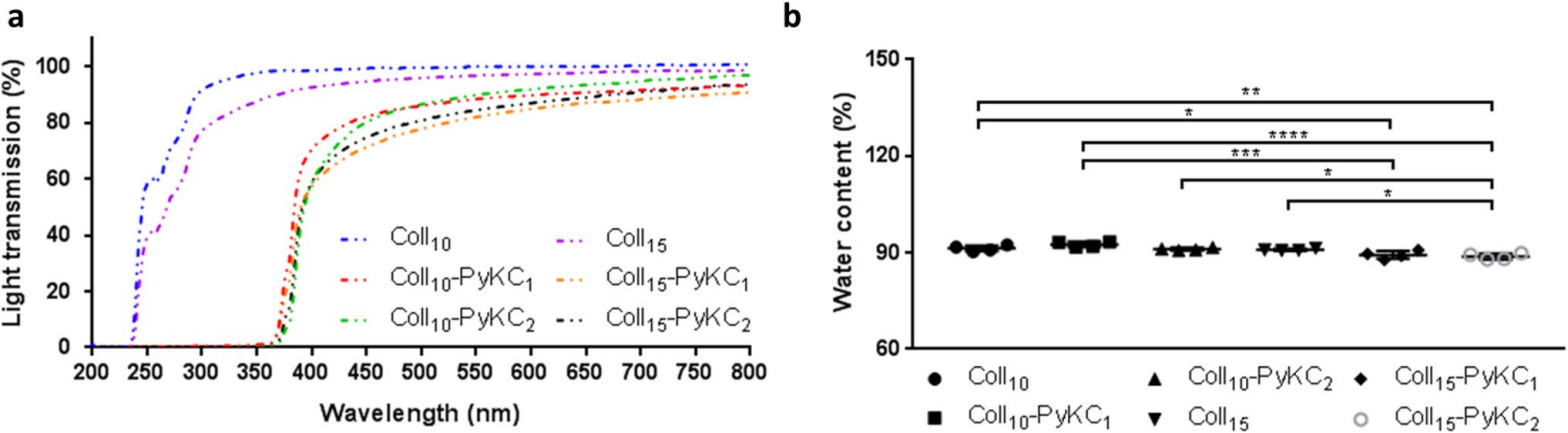
Characterization of crosslinker free Coll-PyKC hydrogels. (**a**) Light transmission measurement through PyKC collagen hydrogels (N = 3) in the wavelength range of 200–800 nm compared with EDC/NHS crosslinked collagen hydrogels. (**d**) The water content of PyKC hydrogels were compared with two control collagen hydrogels (N = 4), Coll_10_ and Coll_15_. A value of *p* < 0.05 was considered statistically significant. *, **, *** and **** represent *p* < 0.05, *p* < 0.01, *p* < 0.001 and *p* < 0.0001, respectively.

The light transmittance of all PyKC hydrogels was similar for all samples (Fig. 2a). Control hydrogels did not block UV light (100–400 nm); however, PyKC containing hydrogels blocked most light in the UV range. The transmittance of the PyKC-containing collagen hydrogel increased with increasing wavelength, similar to the human cornea^31^, and overall results indicated that 10% collagen-containing PyKC gels showed the highest levels of optical transmission (~ 80%) in the visible region (between 400 and 600 nm). This reached ~ 90% at the far visible region (between 600 and 800 nm) for all PyKC-containing hydrogels.

### Water content measurement

Water content of all the hydrogels was ~ 90% (Fig. 2b). Increased PyKC concentration alone did not significantly influence the water content of PyKC containing hydrogels; Coll_10_-PyKC_1_ vs. Coll_10_-PyKC_2_ (*p* = 0.1959), and Coll_15_-PyKC_1_ vs. Coll_15_-PyKC_2_ (*p* = 0.9387). Adding a high concentration of PyKC to highly concentrated collagen decreased the water content compared to control hydrogels; Coll_10_ vs. Coll_15_-PyKC_2_ (*p* = 0.0057), and Coll_15_ vs. Coll_15_-PyKC_2_ (*p* = 0.0248). Collagen concentration alone had no effect on water content; Coll_10_ vs. Coll_15_ (*p* = 0.9788).

### Suture pull-out study

**Figure 3:**
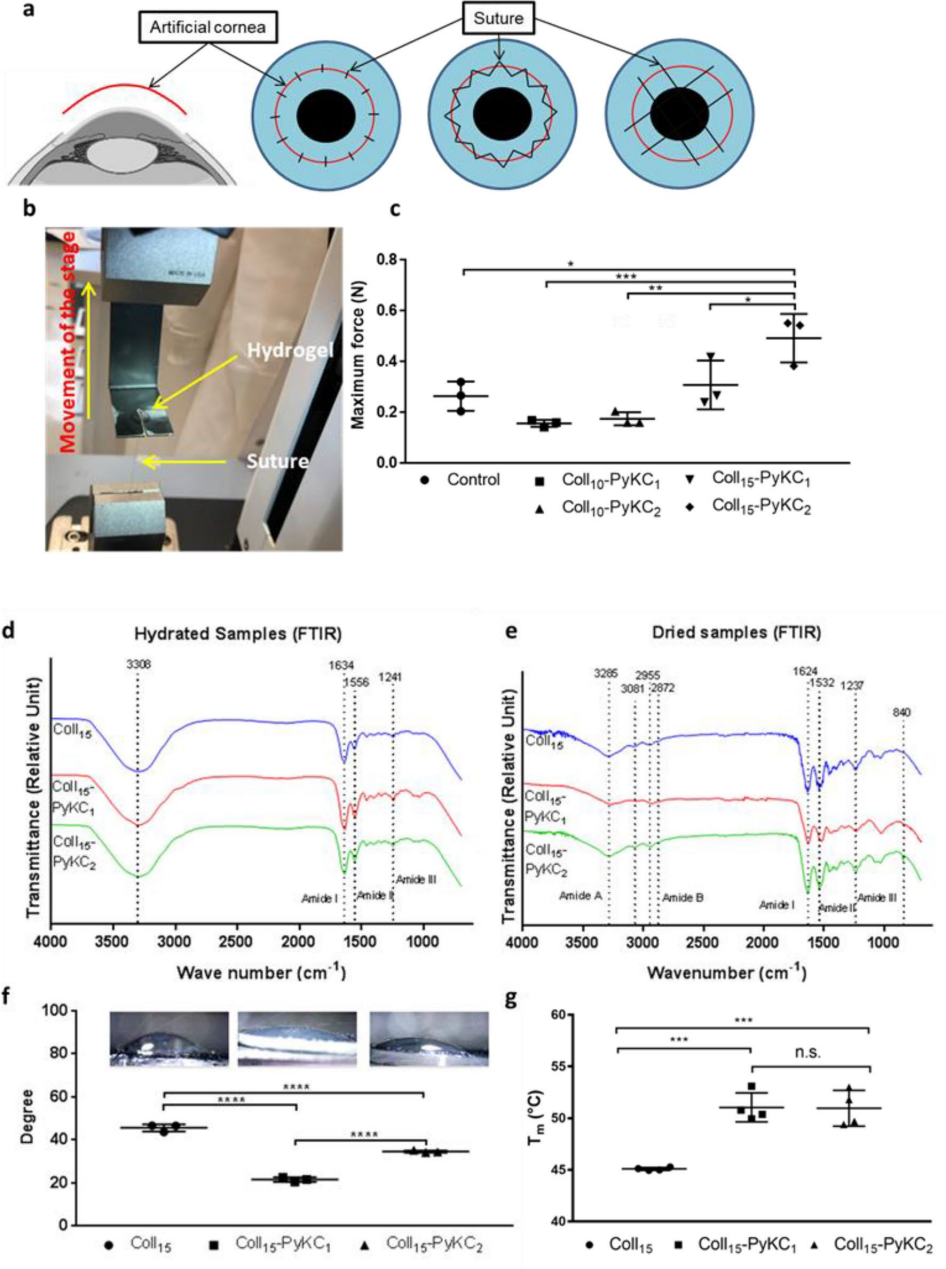
(**a**) Illustration of the importance of the suturability of the corneal implants. After removing the diseased cornea, the artificial cornea can be placed into the host eye with different suturing techniques. Suturing can be done through interrupted, continuous, or overlaying sutures to stabilize and retain the implant. Images were created with BioRender.com on a standard academic license. (**b**) Machine setup for the suture pull-out study. (**c**) Measurement of maximum force the hydrogels (N = 3) withstand during suture pull out study before the suture cut off the materials and comparison was made against control hydrogel (Coll_10_). Characterization of Coll_15_-PyKC_1_ and Coll_15_-PyKC_2_ hydrogels compared with control Coll_15_ hydrogel. FTIR spectra of hydrated (**d**) and dried hydrogel sample (**e**) of PyKC and collagen hydrogel compared with control hydrogel. (**f**) Contact angles measurement on different hydrogels with corresponding micrographs of water contact angles (N = 3). (**g**) The denaturing temperature measurement (Tm) of different hydrogels (N = 4). A value of *p* < 0.05 was considered statistically significant. n.s., *, **, *** and **** represent *p* > 0.05, *p* < 0.05, *p* < 0.01, *p* < 0.001 and *p* < 0.0001, respectively.

The suture pull-out study was designed to be a useful indicator of biomaterial performance during the implantation of an artificial cornea. Ideally, an implant should withstand high mechanical load during suturing before the suture tears through the material. For the suture pull-out study (Fig. 3a–c), Coll_10_ was used as control. The suture resistance of Coll_15_-PyKC_2_ (0.49 ± 0.09 N) was the highest among the tested samples and was significantly higher than that of control and other PyKC hydrogels (Coll_10_, Coll_10_-PyKC_1_, and Coll_10_-PyKC_2_). No difference was observed between Coll_10_-PyKC_1_ and Coll_10_-PyKC_2_ (*p* = 0.9972). No statistically significant difference was noticed for 1% PyKC hydrogel; when the collagen concentration was increased from 10 to 15% for the 1% PyKC hydrogel (Coll_10_-PyKC_1_ vs. Coll_15_-PyKC_1_, *p* = 0.1124). Interestingly, high resistance could be achieved when both PyKC and collagen concentrations were enhanced, for example, Coll_10_-PyKC_1_ vs. Coll_15_-PyKC_2_ (*p* = 0.0008). The results indicate that the Coll_15_-PyKC_2_ can be used as for implantation with interrupted sutures. The difference was also significant when PyKC concentration was increased in high concentration collagen hydrogels, Coll_15_-PyKC_1_ vs. Coll_15_-PyKC_2_ (*p* = 0.0449). Based on the suture pull out study we focused on hydrogels with the highest concentration of collagen while varying the concentration of PyKC.

### Fourier-transform infrared spectroscopy (FTIR)

The FTIR spectra of the three hydrated gels (Fig. 3d) exhibited amide I bands at 1634 cm^-1^, attributed to stretching vibration of C=O bonds coupled to N–H bending vibrations of the polypeptide backbones. In addition, amide II and amide III bands were observed at 1556 cm^-1^ and 1241 cm^-1^ respectively, associated with the N–H bending vibrations coupled to C–N stretching vibrations, and the combination peaks between N–H deformation and C-N stretching vibrations, respectively. The potential amide A and amide B bands were obscured by the presence of a broad band centred at 3308 cm^-1^ characteristic of the O–H stretching vibration mode of water. PyKC containing hydrogels showed similar bands to the collagen control hydrogel.

The dried hydrogel samples (Fig. 3e) showed similar spectral features to the ones in the hydrated state, with slightly shifted band positions. The FTIR spectra showed amide A bands associated with N–H stretching at 3285 cm^-1^, with shoulders at 3081 cm^-1^ corresponding to sp^2^ C–H stretching of aromatic residues. Amide B double bands were observed at 2955 cm^-1^ and 2872 cm^-1^ corresponding to the two stretching modes of CH_2_. Stretching of C=O bonds of the polypeptide backbone was indicated by the presence of the amide I band at 1624 cm^-1^. Amide II and amide III bands at 1532 and 1237 cm^-1^, respectively, were indicative of N–H in plane bending vibrations coupled with C–N and C–H stretching. The remaining signals were assigned as follows: 1456 cm^-1^ O–H bending coupled with C–H scissoring, 1404 cm^-1^ carboxyl O–H bending, and broad band at 1033 cm^-1^ corresponding to C–O stretching. The dried hydrogels induced an additional band at 840 cm^-1^ corresponding to the pyrene ring, which was missing in the control hydrogel. As expected, the intensity of this band was relatively high in 2% PyKC hydrogel. This pyrene signal was obscured in the hydrated gel by the relatively broader band ascribed to the presence of water.

### Contact angle measures

The contact angle is a measure of the wettability of the sample (Fig. 3f). We found significantly higher contact angle values for the Coll_15_ control relative to Coll_15_-PyKC_1_ (*p* < 0.0001) as well as Coll_15_-PyKC_2_ (*p* < 0.0001). The contact angles increased from 21.61 ± 1.08° to 34.59 ± 0.54° as the PyKC amount in the hydrogel was increased from 1 to 2% which indicates the enhanced hydrophobicity of the resultant hydrogel.

### Thermal properties: differential scanning calorimetry (DSC)

Thermal stability was measured through DSC by evaluating the denaturation temperature (Tm; Fig. 3g). Thermal stability increased with PyKC collagen hydrogel content compared with control EDC crosslinked hydrogels [Coll_15_ vs. Coll_15_-PyKC_1_ (*p* = 0.0003) and Coll_15_ vs. Coll_15_-PyKC_2_ (*p* = 0.0003)]. However, the change of T_m_ was not significant when PyKC concentration was increased (Coll_15_-PyKC_1_ vs. Coll_15_-PyKC_2_ (*p* = 0.9955).

### In vitro implant cellular compatibility

**Figure 4:**
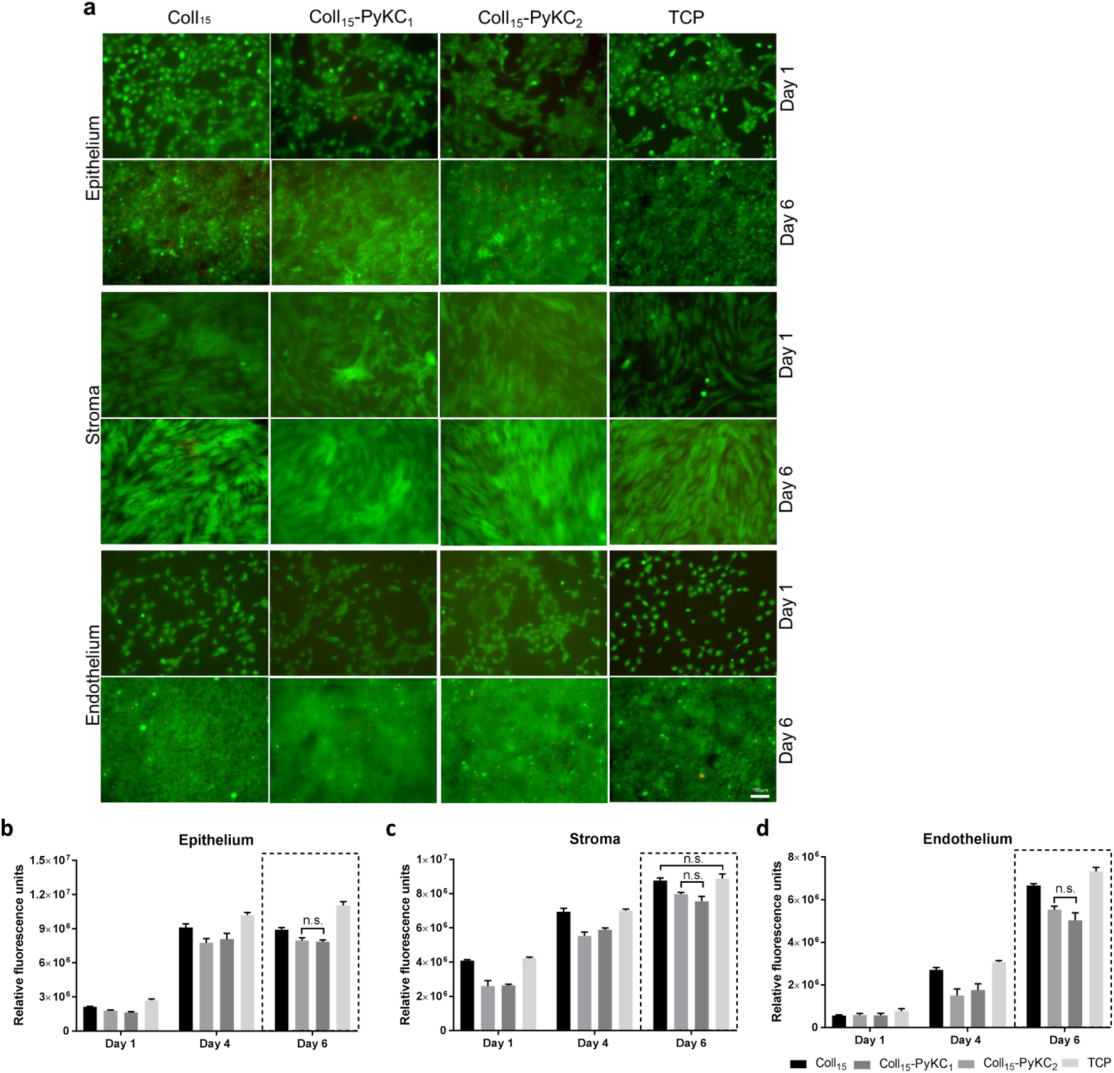
Biocompatibility studies of PyKC containing collagen hydrogels. (**a**) Live/dead pictures of corneal epithelial, stromal, and endothelial cells culture on Coll_15_-PyKC_1_ and Coll_15_-PyKC_2_ hydrogels at day 1 and Day 6. Green and red colour represents live and dead cells, respectively. Scale bar is 100 μm. Rate of proliferation on different hydrogels was evaluated through Alamar blue study at different time points for corneal (**b**) epithelial (**c**) stromal and (**d**) endothelial cells. Statistical analysis was done only on final day (day 6) of the Alamar Blue assay as we wanted to compare the final cell proliferation on different hydrogels and only the non-significant (n.s.) differences among the hydrogels were presented for the simplification of the presentation. For Live/dead and Alamar Blue study, Coll_15_ hydrogel and tissue culture plate (TCP) were used as controls.

The three major corneal cell types, HCEC, HCF, and CEC, were employed to evaluate the biocompatibility of the newly developed hydrogels. The 15% collagen containing PyKC hydrogels was assessed, and Coll_15_ hydrogel and TCP were served as controls. Alamar Blue bioassay was performed to evaluate cell proliferation on the hydrogels, and live/dead staining (Fig. 4a) was used to assess cytotoxicity. Statistical analysis was done on the final day of the Alamar Blue assay and the non-significant differences were presented in the figures (Fig. 4b–d). Cells grown on TCP proliferated the most, for all cell types, and there was no difference in cell growth for any cell type between Coll_15_-PyKC_1_ and Coll_15_-PyKC_2_ hydrogels.

**Figure 5:**
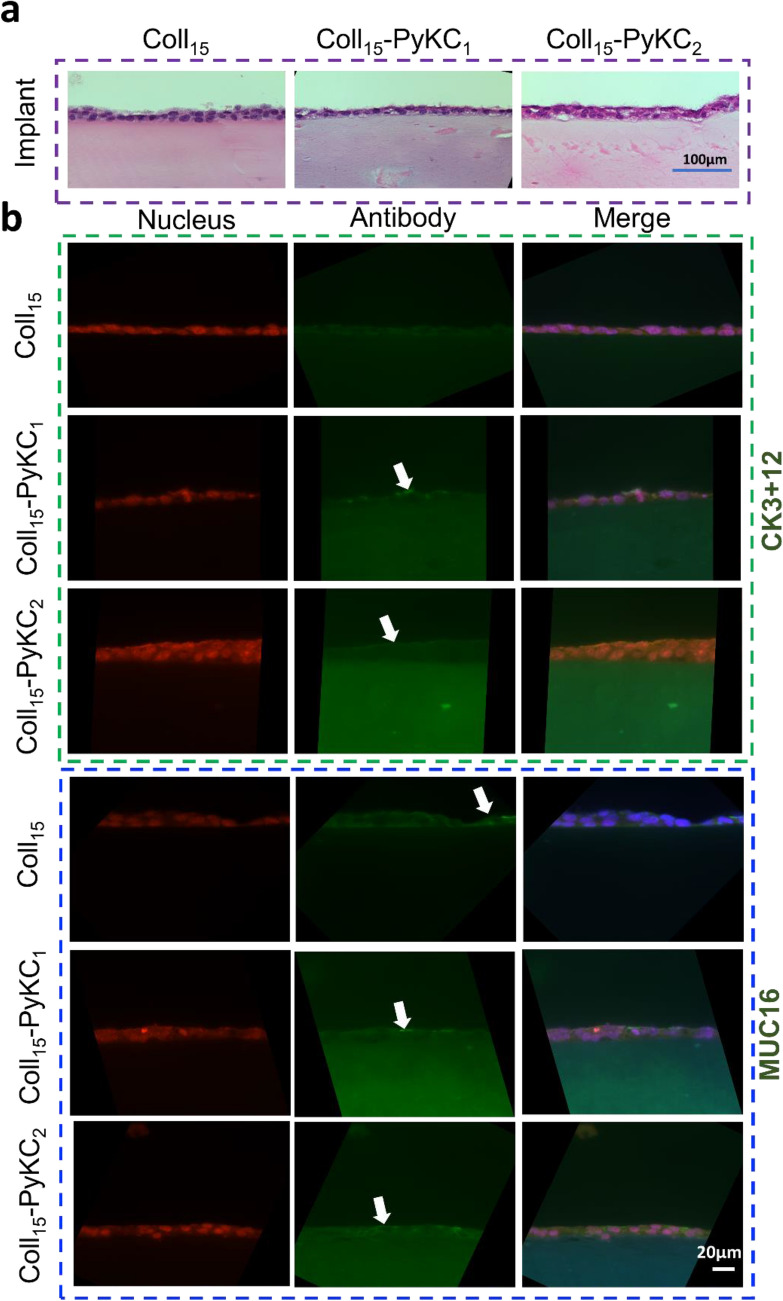

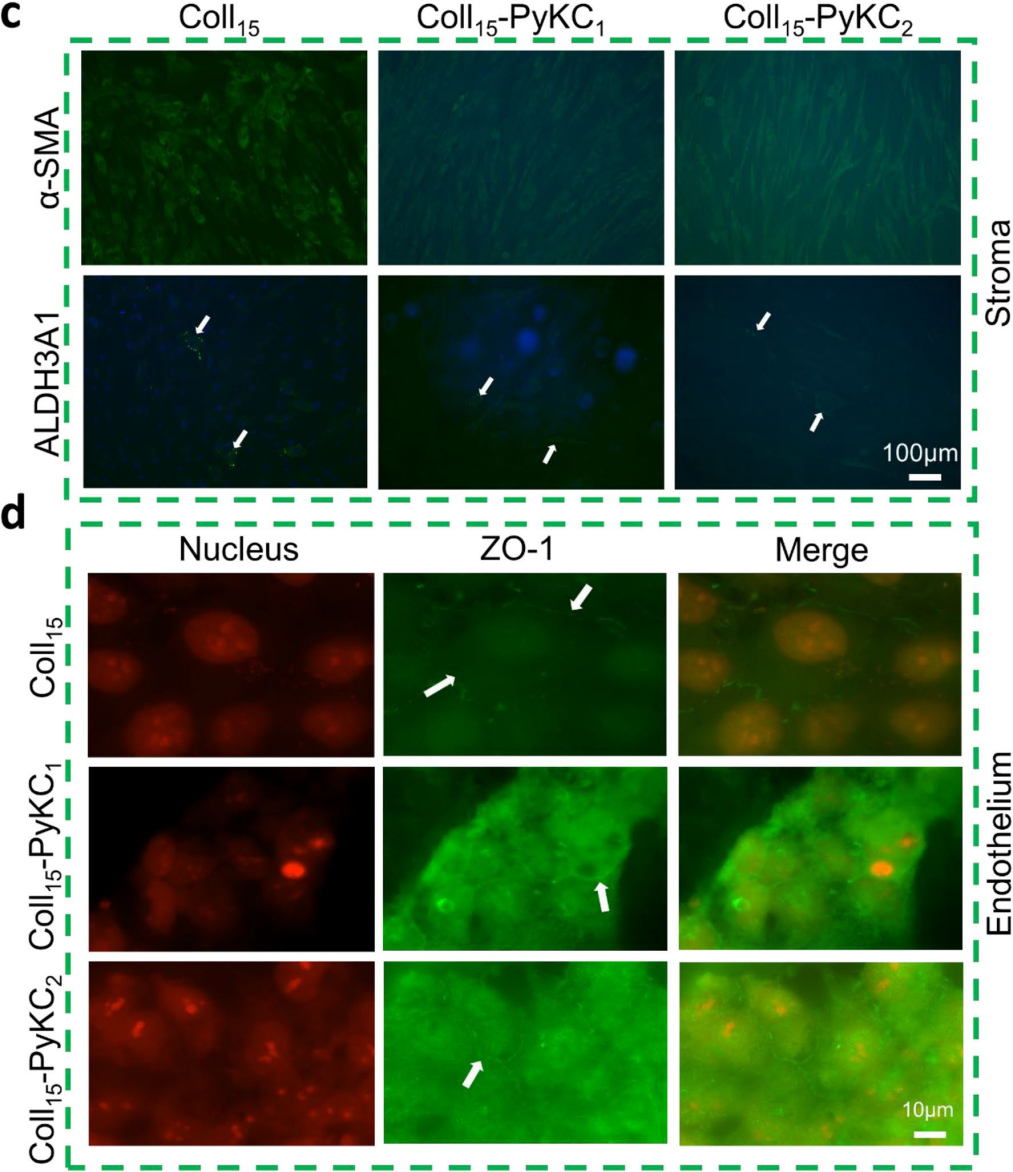
Histological and phenotypic evaluation of corneal cells on Coll_15_-PyKC_1_ and Coll_15_-PyKC_2_ hydrogels. 15% collagen (only) hydrogels (Coll_15_) were used as a control. (**a**) H&E staining of corneal epithelial cells (HCEC) on different 15% collagen containing hydrogels. The scale bar is 100 μm. (**b**) Images from fluorescence microscopy for CK3 + 12 and MUC16 antigen expression by HCEC on different hydrogels. Nuclei are either red from EthD-1 or blue from DAPI staining. Green represents antibody staining (arrows show the position of antibody staining). The right column represents the merged images. The scale bar is 20 μm. (**c**) Staining for α-SMA and ALDH3A1 in primary corneal stromal cells (pHCF) cultured on different hydrogels. The specific antigen is stained for with green fluorescence, and nuclei stained blue with DAPI. Arrows represent relatively few ALDH3A1 positive cells on the different hydrogels. Scale bar is 100 μm. (**d**) ZO-1 staining by corneal endothelial cells cultured on different hydrogels. Red represents nuclei stained with EthD-1 and arrows show antigen expression at the cell membrane. The scale bar is 10 μm.

HCEC growth was prominent on control hydrogels compared to the PyKC hydrogels (Fig. 4b), throughout the study. Alamar Blue assay showed a negligible effect on metabolic activity between Day 4 and Day 6, for all groups. This may be attributed to cell confluence and contact inhibition, which was supported by the live/dead data showing confluence on day 4 of the cultures (Supplementary Fig. 2). H&E images also confirmed a continuous epithelial layer of the top of the hydrogels (Fig. 5a).

HCF growth was similar on Coll_15_ and TCP (Fig. 4c). There was a gradual increase in cell proliferation over time for all groups. Although on day 6 Alamar Blue data showed a substantial difference (Coll_15_ vs. Coll_15_-PyKC_1_ and Coll_15_ vs. Coll_15_-PyKC_2_, significance *p* = 0.0092, *p* = 0.0006, respectively) in cell proliferation between control and PyKC hydrogels, live/dead analysis showed that all the biomaterials along with TCP led to confluence by day 6 of the culture.

The viability of CEC was greater on TCP compared to the other surfaces; TCP is coated with FNC to facilitate cell growth. Due to low cell seeding number, the changes in cell proliferation between days were prominent. On the day 6, more cells were present on control hydrogel than PyKC hydrogels (Coll_15_ vs. Coll_15_-PyKC_1_ and Coll_15_ vs. Coll_15_-PyKC_2_, significance *p* = 0.0010 and *p* < 0.0001, respectively). Although cells were not confluent at day 4 (Supplementary Fig. 2) on any surfaces, the confluence was apparent on all surfaces by day 6 (Fig. 4d).

### Phenotype of corneal cells cultured on hydrogels

Phenotypic evaluation of corneal cells on different hydrogels was performed by immunofluorescence microscopy. HCEC cultured on different PyKC, and control hydrogels (Fig. 5b) expressed cytokeratin 3 + 12 and MUC16. Autofluorescence from PyKC hydrogels compromised the picture quality. pHCF cultured on all hydrogels expressed α-SMA, whereas very few cells expressed the aldehyde dehydrogenase marker, ALDH3A1 protein (Fig. 5c). PyKC hydrogels showed autofluorescence using the blue laser channel. ZO-1 is a marker of the tight junctions. CEC cultured on the different hydrogels expressed ZO-1 antigen at cell–cell junctions (Fig. 5d).

### In vitro evaluation of human adaptive immunity in the presence of the hydrogels

**Figure 6:**
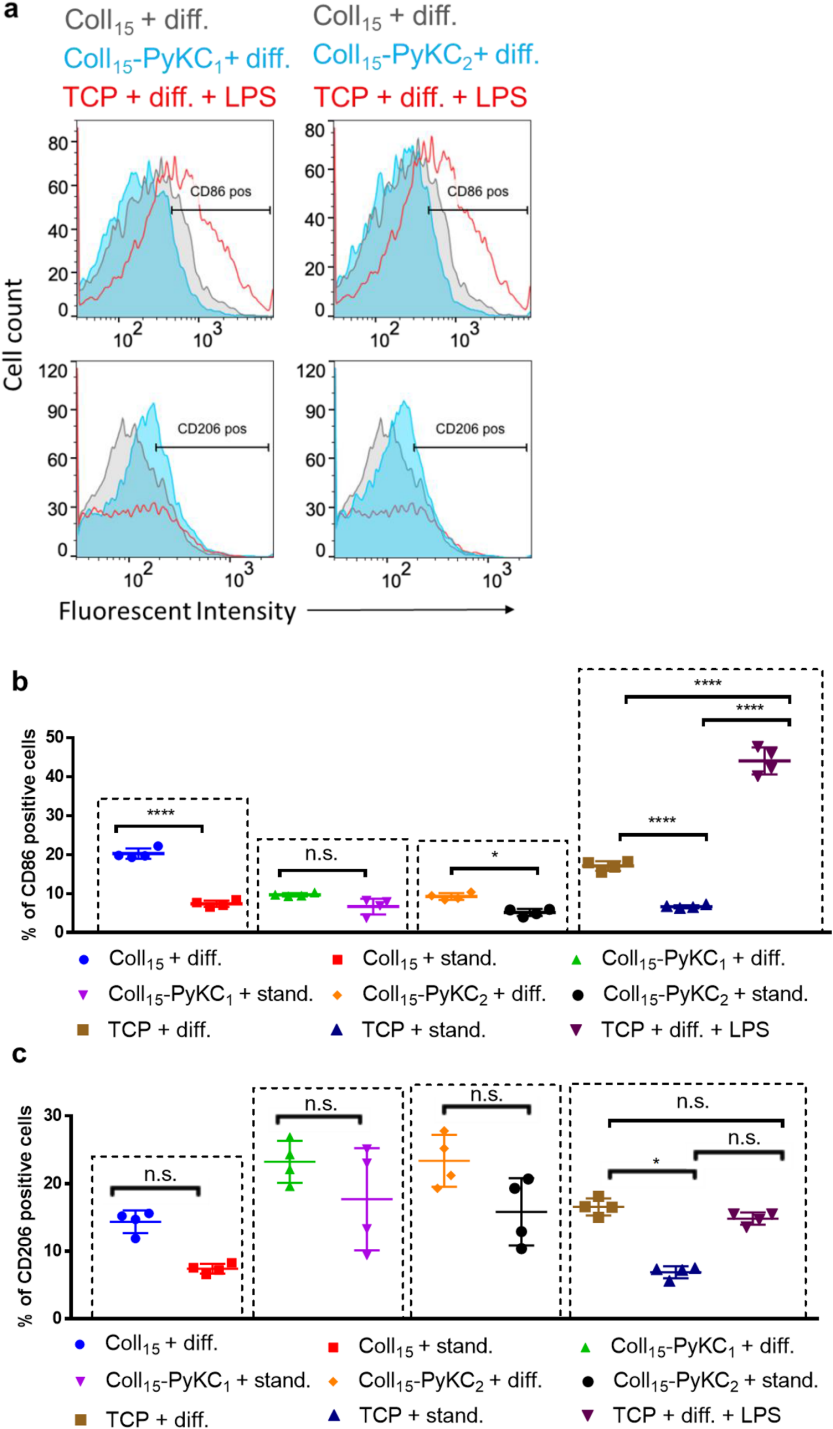
Human monocyte THP-1 cell differentiated to dendritic cells when cultured on different hydrogels. THP-1 cells were cultured with standard media (stand.) or differentiation media (diff.). (**a**) At day 7, the expression of CD86 (pro-inflammatory activated marker) and CD206 (inactivated marker) by cells on two PyKC collagen hydrogels cultured with differentiation media compared with control hydrogels. Cells on TCP with LPS media were used as a positive control. Complete analysis of CD86 positive cells (**b**) and CD206 positive cells (**c**) were evaluated. To simplify the figure, statistics are only shown for differences within the same hydrogel group (or TCP) when treated with different media. Quantitative results were reported as the mean ± S.D. from four independent samples.

We wanted to determine whether the monocytic cell line THP-1 differentiation to the activated dendritic cells (as implied by CD86 expression) or the inactivated dendritic cells (with CD206 expression) phenotype is impacted by culture on the different hydrogels. Morphological changes of the cells were evaluated on day 1 (Supplementary Fig. 3a) and day 7 (Supplementary Fig. 3b). On day 1, round THP-1 cells were observed for all groups except the cells cultured with LPS. This difference became more prominent at day 7. LPS cultured cells on day 7 were elongated in shape, and tightly attached to the surface. Cells treated with differentiation media appeared larger than cells cultured on hydrogels with standard media. To confirm dendritic cell activation, the expressions of CD86 and CD206 were evaluated after 7 days of cell culture on different hydrogels and TCP. Cells cultured with differentiation media with LPS on TCP showed the highest expression of CD86 and lowest expression of CD206 (Fig. 6a), which was the positive control for cell activation. Cells cultured with differentiation media on Coll_15_-PyKC_1_ and Coll_15_-PyKC_2_ showed relatively low expression of CD86 and higher expression of CD206 compared with control Coll_15_ hydrogels (Fig. 6a). CD86 and CD206 expression was shown in Fig. 6b and c, respectively. To simplify the figure, the statistics are shown only for differences within the same hydrogel or TCP to compare the effect of differentiation media versus standard media. Moreover, the control hydrogel generated a relatively high expression of CD86 compared with PyKC hydrogels in the differentiation media (Coll_15_ + diff. vs. Coll_15_-PyKC_1_ + diff. and Coll_15_ + diff. vs. Coll_15_-PyKC_2_ + diff., *p* < 0.0001 and *p* < 0.0001, respectively). CD86 expression by cells on control hydrogels with standard media was statistically similar to that on both PyKC hydrogels. CD86 expression on control hydrogel with standard media was similar to cells on PyKC hydrogels cultured with differentiated media, suggesting that the PyKC hydrogels regulated the inactivated phenotype of the dendritic cells. Interestingly, cells cultured on TCP with differentiation media showed high expression of CD86 compared to PyKC hydrogels (TCP + diff. vs. Coll_15_-PyKC_1_ + diff. and TCP + diff. vs. Coll_15_-PyKC_2_ + diff., *p* < 0.0001 and *p* < 0.0001, respectively). Cells on PyKC hydrogels with differentiation media showed similar expression of CD86 to cells on TCP with standard media. There was no significant difference between the two PyKC collagen hydrogels. Control hydrogels with the differentiation media showed lower expression of CD206 compared with PyKC hydrogels (Coll_15_ + diff. vs. Coll_15_-PyKC_1_ + diff. and Coll_15_ + diff. vs. Coll_15_PyKC_2_ + diff., *p* = 0.0329 and *p* = 0.0286, respectively). CD206 expression increased for cells on all hydrogels with differentiation media, although the differences within groups were not statistically significant. Culture with standard media was associated with greater expression of CD206 for PyKC hydrogels than with control hydrogels or TCP. The complete statistical analysis between groups is presented in the supplementary Table 1.

### Evaluation of preliminary antiviral properties

**Figure 7:**
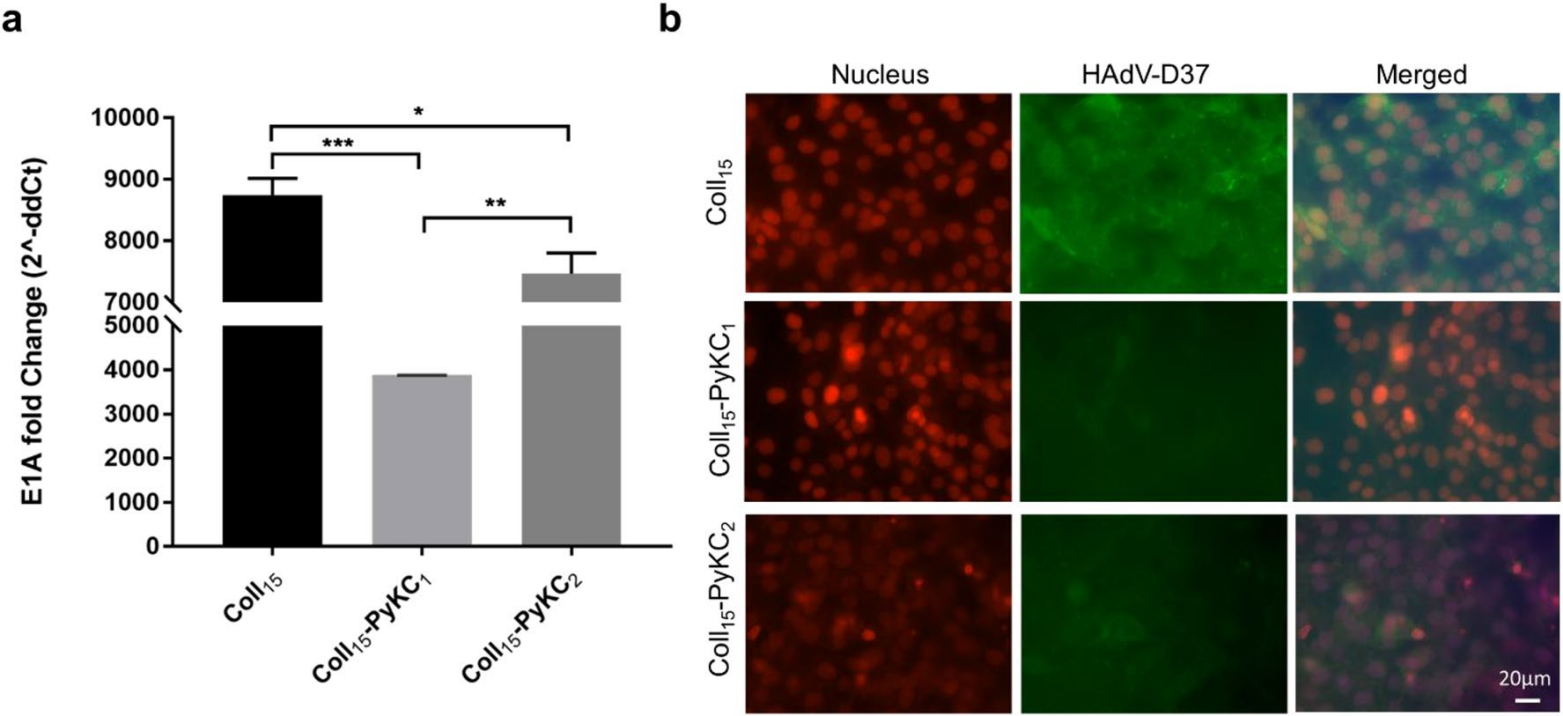
(**a**) Fold changes of the viral E1A gene were evaluated at 24 h post infection hours using real-time PCR. The amount of E1A was significantly less with Coll_15-_PyKC_1_ compared to Coll_15._ Data was normalized to GAPDH and are expressed as the mean ± standard deviation. The quantification of E1A was calculated using the 2^-ddCt method, and the results are presented as relative fold change. A value of p < 0.05 was considered statistically significant. *, **, and *** represent *p* < 0.05, *p* < 0.01, and *p* < 0.001, respectively. (**b**) Immunofluorescence assay. Immunostaining for HAdV-D37 in human corneal epithelial cells (HCECs) cultured on different infected hydrogels. Cells were observed at 48 hpi, green fluorescence indicates the adenovirus load as assessed with anti-Ad5 and secondary antibody conjugated with FITC and red represents nuclei stained with EthD-1. The scale bar is 20 μm.

To determine any effect of the hydrogels on resistance to viral infection, we grew HCECs on the different hydrogels and infected them with HAdV-D37. Viral DNA replication was evaluated by infecting hydrogel-cell constructs with HAdV-D37 at MOI 1. DNA was isolated from the infected and control constructs at 24 h post infection (hpi) for qRT-PCR. Compared to infected Coll_15_ cells, the quantity of E1A DNA was lowest in Coll_15_-PyKC_1_ cells, followed by Coll_15_-PyKC_2_ cells. The relative fold change of HAdV-D37 DNA is depicted in Fig. 7a. The analysis through immunofluorescence assay also revealed a reduction in HAdV-D37 in the Coll_15_-PyKC_1_ group compared to the control Coll_15_. Notably, fluorescence was less in the construct Coll_15_-PyKC_1_, followed by Coll_15_-PyKC_2_ at 48 hpi (Fig. 7b). Thus, the measurements of viral DNA were consistent with the immunofluorescence results, which exhibited the same inhibition profile.

## Discussion

Cornea transplantation is one of the most common forms of organ transplantation. As the outermost part of the eye, the cornea plays a critical role in vision by focusing and transmitting light to the retina and protecting the eye from external infectious agents. Corneal damage is one of the prime reasons for blindness globally, and the primary treatment is a replacement of the diseased cornea with a donor corneal allograft. However donor corneas remain scarce worldwide, resulting in many patients left untreated^32^. To fill the gap between supply and demand, much research has focused on developing an artificial cornea. Toward this end, CACs have been transplanted into humans^13,15^, in most cases they consisted of collagen crosslinked with EDC and NHS. Carbodiimide-based chemical crosslinkers, such as EDC-based methods are considered a standard strategy with low cytotoxicity, but the mechanical strength of crosslinked collagen is less than satisfactory. EDC crosslinks free primary amino groups of lysine residues with cell-reactive carboxylate anions on glutamate or aspartate residues. This may reduce the accessibility of essential cell attachment points on collagen-based biomaterials^33^. Carbodiimide forms zero-length crosslinking that very quickly produces rigid bonding. Shorter crosslinking times make the implants inhomogeneous, susceptible to microcracks and shearing^13^.

In a clinical study with chemically crosslinked collagen based implants, overlying sutures crossing the centre of the cornea were used to avoid implant extrusion^13^. These sutures impeded epithelial growth onto the centre, leaving the implant susceptible to collagenases and generating thinning, haze and astigmatism^13,34^. Other crosslinkers, e.g., glutaraldehyde and hexamethylene diisocyanate, have also been used to produce collagen-based implants; however, modification of important functional groups of the parent biomaterial and low biocompatibility remain limitations. Recently, light-induced crosslinking of ECM components in the presence of photo initiators has become popular^29,35^, although patients with corneal inflammation are highly light-sensitive and it can be difficult for them to tolerate intense visible light application for any length of time without anaesthesia^20^.

Any biomaterials designed for use in an artificial cornea should permit transparency similar to that of the human cornea. All the CACs previously developed were transparent from UV to visible light^13,15^ as we observed for the control hydrogel. However, control EDC/NHS crosslinked hydrogels cannot block UV light, but PyKC hydrogels can, while still permissive of transmission of visible light. The human cornea blocks some UV light^31^. Therefore, patients implanted with PyKC hydrogels would not require additional protection from UV light, as is recommended for CAC implants^13^. The blocking of UV light could be ascribed to the presence of pyrene rings in PyKC. Pyrene is the polycyclic aromatic hydrocarbon with high symmetrical properties. Pyrene is one of the most studied organic molecules in terms of its photophysical properties. Pyrene has 16 π electrons, but however, does not follow the Hückel’s law of aromatic compounds^36^. This electronic arrangement makes it an interesting compound to evaluate in host–guest systems where two or more molecules combine in special structural relationships. Among the numerous techniques employed to study host–guest chemistry, UV-vis spectroscopy is one of the most frequently used techniques^37^. As pyrene exhibits characteristic absorption spectra in the UV-region, pyrene UV absorption spectra was used to determine the critical micellar concentration in different surfactant solutions^38^. Because of its highly conjugated nature as an aromatic molecule, pyrene has several absorption bands extending from 200 to 360 nm^39^. The equilibrium water content of the hydrogels was measured to ensure uniformity^15^. All the hydrogels contained similar water content of around 90% which is greater than the water content of human corneas (78%)^40^. Collagen-based CACs are vulnerable to enzymatic digestion, and this makes them unsuitable for implantation in patients with inflammatory corneal diseases. Collagenase solution (5 U/mL) was also used to evaluate the properties of porcine and recombinant human collagen matrices for optically clear tissue engineering applications^41^. This same concentration has been used as a standard in earlier reports on biomaterial development for artificial corneas^16,42^. However, 5 U/mL is considerably more concentrated than in some other published studies where biomaterials persisted over many days instead of hours^43,44^. In a study with identical collagenase concentrations^16^, EDC/NHS hydrogels broke down within few hours, as we also observed for our control hydrogels. However, PyKC-containing hydrogels remained stable at the end of the study (28 h) (Supplementary Fig. 1a). The stability against degradation was attributed to the unavailability of breakable bonds of the PyKC hydrogel and its exceptional compartmentalizing ability through tight packing of the nano-fibres, which create a protective envelope for proteins from denaturation^21^. As EDC/NHS treatment induces zero-length crosslinking (does not take part in linkage), they generate rigid hydrogels with a high compressive modulus, which we also observed for the control hydrogels. Increasing concentrations of both PyKC and collagen in the hydrogel increased the compressive modulus (Supplementary Fig. 1b).

Suturability is also critical to translation of biomaterials to corneal implants, and the lack of suturability contributed to the less than the optimal clinical outcome of previously implanted CACs^13^. Herein, we found that high concentrations of both PyKC and collagen contributed to improved suturability but acknowledge that even PyKC hydrogels cannot yet compare in suturability to the native human cornea, which remains intact even when the pulling force is sufficient to break the suture^45^. FTIR confirmed the presence of pyrene unit in the hydrogel, as pyrene showed characteristic aromatic stretching near 840 cm^-1^ ^46^. We also evaluated wettability of the hydrogel surfaces by measuring the contact angles^28^. A high contact angle indicates low wettability or a hydrophobic solid surface and a low contact angle indicates high wettability or a hydrophilic surface^47^. It is generally assumed that hydrophilic biomaterials result in the most optimal biocompatibility^48,49^, however, there are exceptions^47^. Biomaterial surface interactions for extremely hydrophilic surfaces may trap cells by high cell-substrate adhesive forces, and this could reduce cell–cell communication and monolayer confluence^50^. On the other hand, highly hydrophobic surfaces will exhibit less protein interactions, which can prevent or reduce cell adhesion^51^. In parallel, moderately wettable (contact angles between 48 and 62°) surfaces appeared to be optimal for cell adhesion and growth^52^. Our control hydrogel contact angle was close to a moderately wettable surface and was superior for corneal cell proliferation. We observed that the contact angle was reduced by the addition of PyKC, but then increased as the concentration of PyKC was increased. Regardless, for the PyKC hydrogels, wettability did not influence much on the biocompatibility of the hydrogels as shown by cell proliferation and the live/dead assays. It was previously shown that contact angles have a limited impact on biocompatibility when corneal endothelial cells are cultured on hydrogels^53^. We previously showed also that an increasing contact angle did not influence the recellularization of the decellularized porcine cornea^28^. We also measure the T_m_ of the hydrogels to evaluate their thermal stability. The heating of hydrogels leads to the denaturation of the native triple helix structure of collagen and converts the triple helix into a single-chain random coil conformation^16^. The T_m_ depends on the chemical nature and the degree of crosslinking. High T_m_ is desirable as shown for human cornea (65 °C)^54^. Although none of our hydrogels reached that level, PyKC hydrogels showed higher T_m_ (50–51 °C), while the control hydrogels showed similar denaturation temperature to a previously published report (~ 46.8)^16^. Improved thermal stability for PyKC hydrogels suggests that PyKC protects entrapped protein/peptide from the heat^21^.

We did not observe any degradation or contraction of the materials unless we added collagenase to the solution. Cells had no negative effect on the materials. Cytotoxicity studies make clear that PyKC containing hydrogels are not cytotoxic to corneal cells. Collagen-based hydrogels have been previously shown to promote corneal cell growth over a long time, as previously published^16,22^, and adding PyKC did not diminish their superior biocompatibility. In addition, phenotypic evaluation showed that HCEC expressed corneal epithelial specific CK3 + 12 and MUC16; however, autofluorescence of the biomaterials compromised the quality of the images for HCECs as was seen with re-cellularized porcine corneas^28^. Furthermore, HCECs cultured on all hydrogels expressed the HCEC-specific marker zonula occludens-1 (ZO-1)^55^. Primary corneal stromal cells showed expression of α-SMA on all the hydrogels. pHCF are normally quiescent and express the unique markers aldehyde dehydrogenases and keratocan. In serum culture, like our culture condition, they proliferate and become stromal fibroblasts while losing their characteristic phenotype^56^. It was shown previously that cultured primary fibroblasts develop a myofibroblast phenotype even on TCP^57^. It has also been shown that when primary corneal fibroblasts are cultured in a collagen matrix, α-SMA expression increased from day 0 to 7 but then decreased from day 7 to 14^58^. It was found that nearly 100% of fibroblasts seeded in a collagen sponge expressed α-SMA^59^. As expected, given that α-SMA expression is typically inversely proportional to that of the fibroblast specific antigen, ALHD3A1, we observed very low ALHD3A1 expression on hydrogel cultured cells. Regardless, in human studies of implanted CACs^13^, they remained mostly transparent^60^. Once corneal wound remodelling is complete, myofibroblasts are known to disappear from the wound space through apoptosis^61^.

Silencing adaptive immunity considered one of the major factors determining graft success. Antigen presenting cells (such as dendritic cells) play important roles in responding to foreign bodies by activating adaptive immunity as well as maintaining immune tolerance. Due to this unique nature APCs can be a useful tools to evaluate the inflammatory responses of the biomaterials^62^. It was shown previously that that human monocytic (THP-1) cell lines can be differentiated rapidly into dendritic cells^63^. In the absence of inflammatory stimulus, antigen-presenting dendritic cells remain immature; however, inflammation causes activation and functional transformation of inactive dendritic cells into mature dendritic cells^64^. In this work we used THP-1 cell line to generate dendritic cells and LPS was used as positive control of activated dendritic cells. We monitored their expression of CD86 and CD206 to determine their response to different hydrogels. The expression of the CD206 has been regarded a differentiation hallmark of immature dendritic cells whereas monocytes and activated dendritic cells do not express CD206^65^. Though immature dendritic cells also express lower levels of CD86^66^. In contrast, activated dendritic cells expressed prominent CD86 to initiate the adaptive immunity response against the graft^66^. We compared standard media with media containing differentiation inducers, specifically rhIL-4 and rhGM-CSF^63^. We used LPS in the study as a positive control as LPS is known to activate APCs^67^. We found that CD86 expression was down regulated when cells were cultured on PyKC hydrogels as compared to controls, to lower expression levels than even on TCP, suggesting that the PyKC hydrogels did not influence differentiation of the THP-1 cells to activated APCs. To the contrary, we observed heightened expression of CD206 by cells cultured on PyKC hydrogels. It was previously shown that CD206 expression was increased by APCs when cultured on highly biocompatible biomaterials^20^. From our results, we suggest that PyKC hydrogels would perform better if implanted in the corneas of diseased eyes and not contribute to inflammation triggered by adaptive immunity.

Ocular surface infections by human adenovirus are common, and the most frequent cause of viral conjunctivitis^68^. Corneal infection is also an important cause of allograft rejection^69^. PyKC has a polycationic backbone which is usually antimicrobial in nature. Therefore, we evaluated the antiviral behavior of the HCEC-covered implants against human adenovirus. Interestingly, PyKC containing implants appeared resistant to infection. This preliminary study supports the idea that PyKC may provide additional protection from viral infection after implantation.

## Conclusion

We have shown that collagen-based artificial corneas can be made without using crosslinkers. Enhanced suturing ability and resistance to enzymatic degradation were improved compared to a standard collagen hydrogel, suggesting potential as an artificial cornea without modification of extracellular matrix component and without compromise of its biocompatibility. As the gelation process is not immediate, homogeneous semisolid mixture could be applied directly at the target site to patch defects in potentially any living body organ, in contrast to hydrogels with crosslinkers which solidify immediately and are cytotoxic. Crosslinker-free PyKC-based hydrogels may show utility as a biological glue and sealant for corneal defects and perforation. Our self-assembled PyKC peptide combined with collagen, gelatin, or other ECM, could be applied directly to any wounded area and once gelation has occurred, would protect, and enable regeneration. This approach can also be utilized to embed and encapsulate—for delivery—drugs, growth factors, and antibodies.

## Supplementary Information

Supplementary Information.

## Data Availability

The datasets used and/or analysed during the current study available from the corresponding author on reasonable request.
