# Supplementary material for: Crosslinker-free collagen gelation for corneal regeneration
Source: Sci Rep. 2022 Jun 1;12:9108. doi: 10.1038/s41598-022-13146-9 (PMC9160259; doi:10.1038/s41598-022-13146-9)
Supplement: Supplementary file 1 — Supplementary Information. [file 41598_2022_13146_MOESM1_ESM.docx]

**Crosslinker free collagen gelation for corneal regeneration**

Mohammad Mirazul Islam^1^, Alexandru Chivu^2^, Dina B. AbuSamra^1^, Amrita Saha^1^, Sumit Chowdhuri^3^, Bapan Pramanik^4^, Claes H. Dohlman^1^, Debapratim Das^3^, Pablo Argüeso^1^, Jaya Rajaiya^1^, Hirak K Patra^2,*^, James Chodosh^1,*^

^1^Massachusetts Eye and Ear and Schepens Eye Research Institute, Department of Ophthalmology, Harvard Medical School, Boston, MA 02114, USA.

^2^Department of Surgical Biotechnology, University College London, London NW3 2PF, UK.

^3^Department of Chemistry, Indian Institute of Technology Guwahati, Assam 781039, India

^4^Department of Chemistry, Ben Gurion University of the Negev, Be’er Sheva, Israel

*Co-corresponding authors: hirak.patra@ucl.ac.uk (Dr. H. K. Patra)

james_chodosh@meei.harvard.edu (Dr. J. Chodosh)

**Supplementary Methods:**

***In vitro* biodegradation.** The resistance of the hydrogels against collagenase digestion was determined according to our published protocol^1^. In brief, each hydrogel (N=3) was placed in a vial containing 5 U/mL collagenase from Clostridium histolyticum (Sigma-Aldrich, St.Louis, USA) in 0.1 M Tris-HCl (pH 7.4) and 5 mM CaCl_2_ at 37°C. The collagenase solution was replaced every 8 h and the percent residual mass of the hydrogel was measured at different time intervals.

**Mechanical characterization.** Mechanical testing (compression) of the hydrogels was conducted using a mechanical tester (Mark-10 ESM 303, Copiague, NY). To perform the compression test, cylindrical samples (diameter=6.0 mm, and thickness=0.5 mm, N=3) were placed in the mechanical tester and measurement was performed with a crosshead speed of 0.5 mm/minute. The compressive stress was recorded as a function of the strain. The resulting stress/strain curve was used to calculate the compressive modulus of each hydrogel.

**Supplementary Result:**

***In vitro* biodegradation.** Enzymatic stability of the PyKC hydrogels was evaluated against high concentration (5 U/mL) of collagenase solution with EDC/NHS crosslinked collagen as a control (Fig. Supplementary Figure 1**a**). Control hydrogel degraded within 4 h, compared to PyKC hydrogels which remained intact at the end of the study (28 h). More than 30% of Coll_10_-PyKC_1_ hydrogels were stable at the end of the study; however, the Coll_10_-PyKC_2_ hydrogels were ~80% stable after 28 h. Only one collagen concentration (10%) was used in the study to show the pattern of the degradation of the hydrogels.

**Mechanical characterization.** The compressive modulus was determined as a mechanical property of the hydrogels with Coll_10_ hydrogels as a control (Fig. Supplementary Figure 1**b**). Stronger materials exhibit a higher compressive modulus. Overall, control hydrogels were mechanically stronger than the other hydrogels; however, the difference between control and Coll_15_-PyKC_2_ hydrogel was not significant (*p* = 0.0555). Coll_10_-PyKC_2_ hydrogels were significantly stronger than Coll_15_PyKC_1_ hydrogels (*p* = 0.0453). Both 2% PyKC hydrogels (Coll_10_-PyKC_2_ vs. Coll_15_-PyKC_2_) exhibited similar mechanical strengths (*p* > 0.9999).


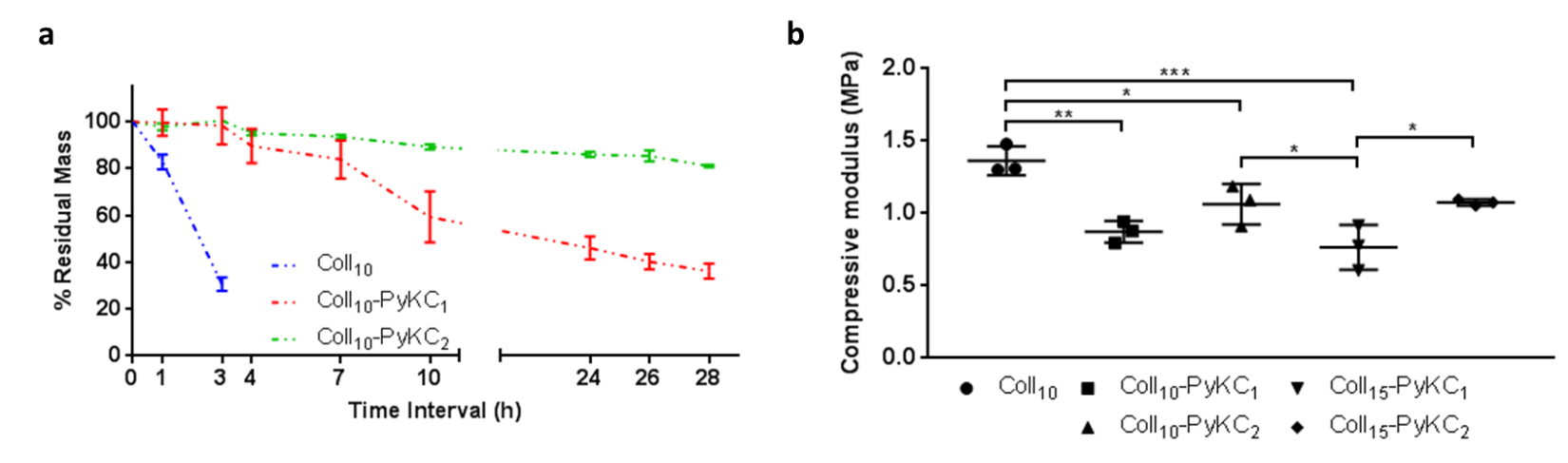
**Supplementary Figure 1:**

**Figure 1:** Characterization of crosslinker free Coll-PyKC hydrogel. (**a**) *In vitro* biodegradation of PyKC hydrogels in high concentrations of collagenase solution in comparison with control collagen (Coll_10_) hydrogel. (**b**) Mechanical property assessment of the PyKC hydrogels, compared with Coll_10_ hydrogel. A value of p < 0.05 was considered statistically significant. *, **, *** and **** represent p < 0.05, p < 0.01, p < 0.001 and p < 0.0001, respectively.

**Supplementary Figure 2:**


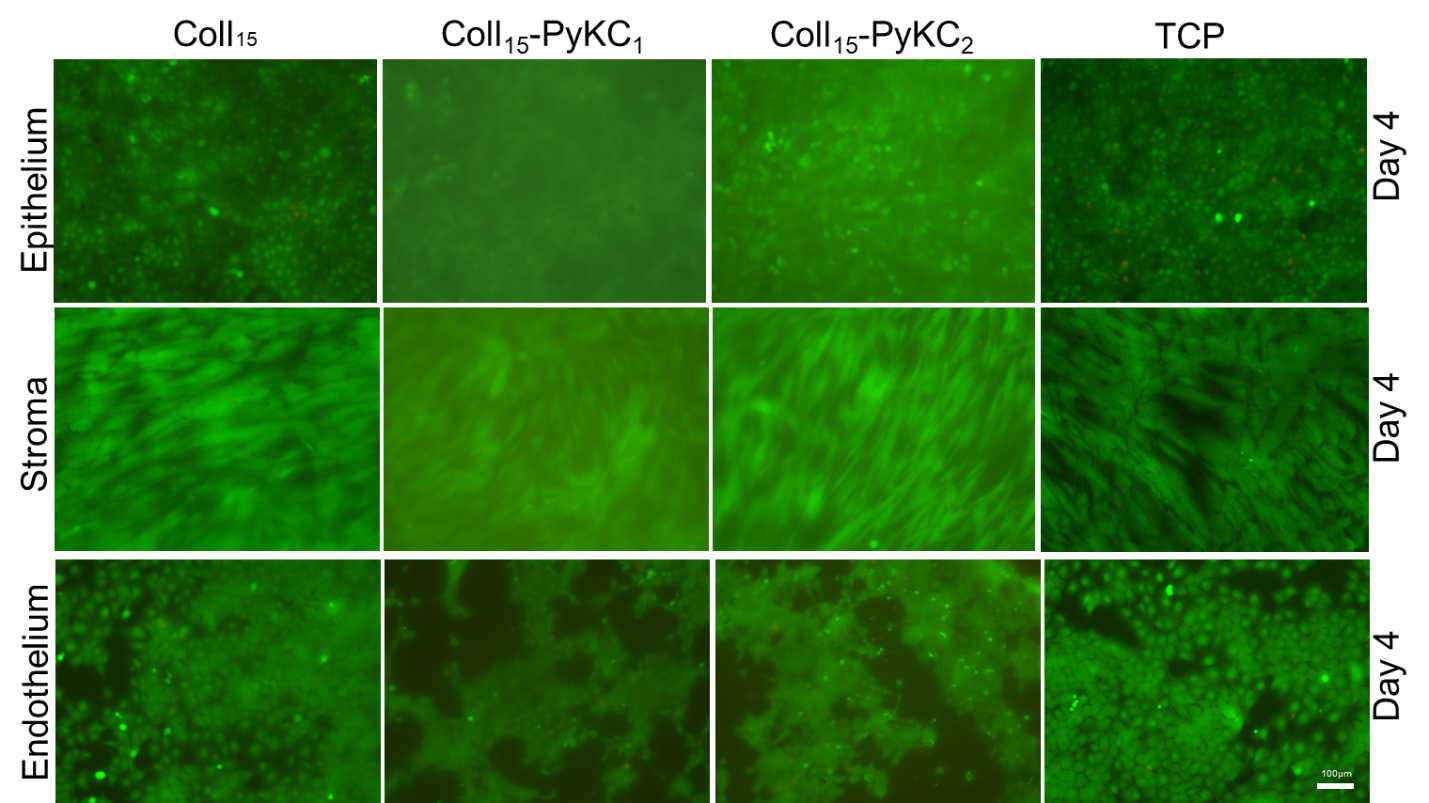


**Figure 2:** Biocompatibility studies of PyKC containing collagen hydrogels. Live/dead pictures of corneal epithelial, stromal, and endothelial cells culture on Coll_15_-PyKC_1_ and Coll_15_-PyKC_2_ hydrogels at day 4. Green and red represent live and dead cells, respectively. Scale bar is 100μm.

**Supplementary Figure 3:**

**
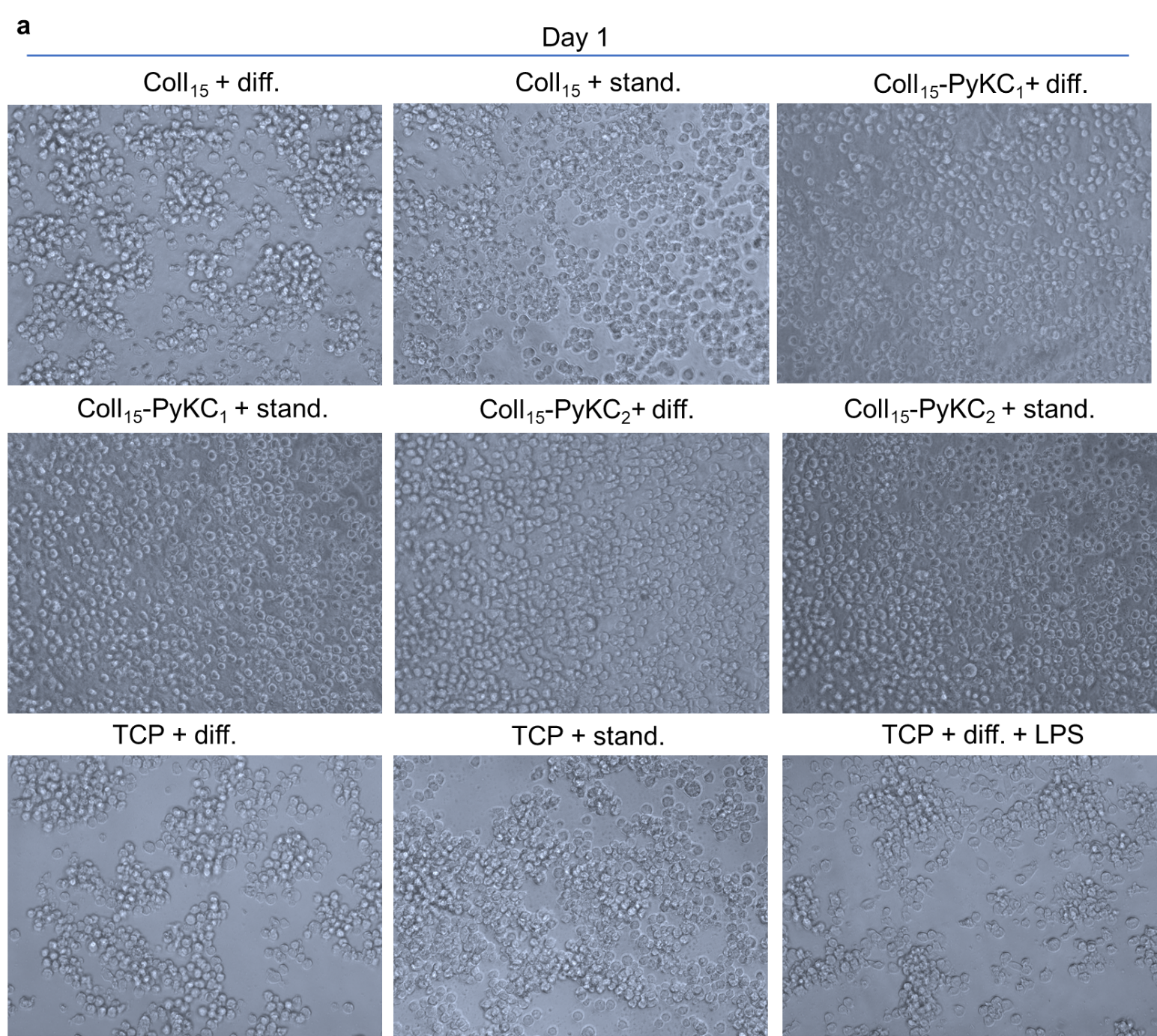
**


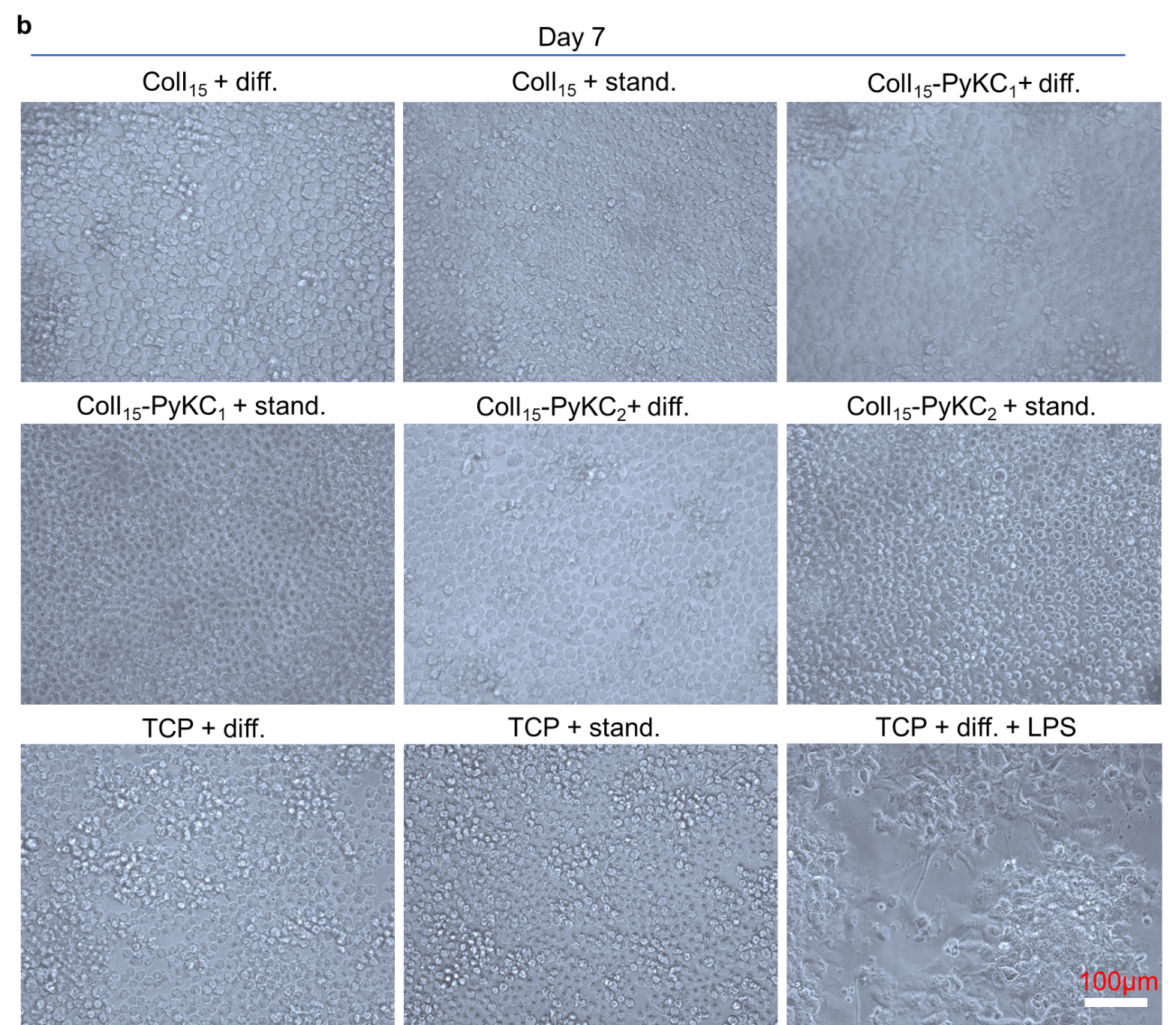


**Figure 3:** Human monocyte THP-1 cell differentiated to dendritic cells when cultured on different hydrogels. Morphology of the THP-1 cells cultured at day 1 (**a**) and day 7 (**b**). Scale bar is 100μm and all the component images are with same magnification.

**Supplementary Table 1:**

*CD86 comparison*

| Tukey's multiple comparisons test | Significant? | Summary | Adjusted P Value |
| --- | --- | --- | --- |
|  |  |  |  |
| Coll_15_ with Differentiation Media vs. Coll_15_ with Standard Media | Yes | **** | <0.0001 |
| Coll_15_ with Differentiation Media vs. Coll_15_-PyKC_1_ with Differentiation Media | Yes | **** | <0.0001 |
| Coll_15_ with Differentiation Media vs. Coll_15_-PyKC_1_ with Standard Media | Yes | **** | <0.0001 |
| Coll_15_ with Differentiation Media vs. Coll_15_-PyKC_2_ with Differentiation Media | Yes | **** | <0.0001 |
| Coll_15_ with Differentiation Media vs. Coll_15_-PyKC_2_ with Standard Media | Yes | **** | <0.0001 |
| Coll_15_ with Differentiation Media vs. TCP with Differentiation Media | No | ns | 0.1438 |
| Coll_15_ with Differentiation Media vs. TCP with Standard Media | Yes | **** | <0.0001 |
| Coll_15_ with Differentiation Media vs. TCP with Differentiation Media +LPS | Yes | **** | <0.0001 |
| Coll_15_ with Standard Media vs. Coll_15_-PyKC_1_ with Differentiation Media | No | ns | 0.5175 |
| Coll_15_ with Standard Media vs. Coll_15_-PyKC_1_ with Standard Media | No | ns | 0.9986 |
| Coll_15_ with Standard Media vs. Coll_15_-PyKC_2_ with Differentiation Media | No | ns | 0.7566 |
| Coll_15_ with Standard Media vs. Coll_15_-PyKC_2_ with Standard Media | No | ns | 0.5275 |
| Coll_15_ with Standard Media vs. TCP with Differentiation Media | Yes | **** | <0.0001 |
| Coll_15_ with Standard Media vs. TCP with Standard Media | No | ns | 0.9981 |
| Coll_15_ with Standard Media vs. TCP with Differentiation Media +LPS | Yes | **** | <0.0001 |
| Coll_15_-PyKC_1_ with Differentiation Media vs. Coll_15_-PyKC_1_ with Standard Media | No | ns | 0.1792 |
| Coll_15_-PyKC_1_ with Differentiation Media vs. Coll_15_-PyKC_2_ with Differentiation Media | No | ns | >0.9999 |
| Coll_15_-PyKC_1_ with Differentiation Media vs. Coll_15_-PyKC_2_ with Standard Media | Yes | ** | 0.0086 |
| Coll_15_-PyKC_1_ with Differentiation Media vs. TCP with Differentiation Media | Yes | **** | <0.0001 |
| Coll_15_-PyKC_1_ with Differentiation Media vs. TCP with Standard Media | No | ns | 0.1681 |
| Coll_15_-PyKC_1_ with Differentiation Media vs. TCP with Differentiation Media +LPS | Yes | **** | <0.0001 |
| Coll_15_-PyKC_1_ with Standard Media vs. Coll_15_-PyKC_2_ with Differentiation Media | No | ns | 0.3459 |
| Coll_15_-PyKC_1_ with Standard Media vs. Coll_15_-PyKC_2_ with Standard Media | No | ns | 0.9021 |
| Coll_15_-PyKC_1_ with Standard Media vs. TCP with Differentiation Media | Yes | **** | <0.0001 |
| Coll_15_-PyKC_1_ with Standard Media vs. TCP with Standard Media | No | ns | >0.9999 |
| Coll_15_-PyKC_1_ with Standard Media vs. TCP with Differentiation Media +LPS | Yes | **** | <0.0001 |
| Coll_15_-PyKC_2_ with Differentiation Media vs. Coll_15_-PyKC_2_ with Standard Media | Yes | * | 0.0221 |
| Coll_15_-PyKC_2_ with Differentiation Media vs. TCP with Differentiation Media | Yes | **** | <0.0001 |
| Coll_15_-PyKC_2_ with Differentiation Media vs. TCP with Standard Media | No | ns | 0.3282 |
| Coll_15_-PyKC_2_ with Differentiation Media vs. TCP with Differentiation Media +LPS | Yes | **** | <0.0001 |
| Coll_15_-PyKC_2_ with Standard Media vs. TCP with Differentiation Media | Yes | **** | <0.0001 |
| Coll_15_-PyKC_2_ with Standard Media vs. TCP with Standard Media | No | ns | 0.9138 |
| Coll_15_-PyKC_2_ with Standard Media vs. TCP with Differentiation Media + LPS | Yes | **** | <0.0001 |
| TCP Differentiation with Media vs. TCP with Standard Media | Yes | **** | <0.0001 |
| TCP with Differentiation Media vs. TCP with Differentiation Media + LPS | Yes | **** | <0.0001 |
| TCP with Standard Media vs. TCP with Differentiation Media + LPS | Yes | **** | <0.0001 |

*CD206 comparison*

| Tukey's multiple comparisons test | Significant? | Summary | Adjusted P Value |
| --- | --- | --- | --- |
|  |  |  |  |
| Coll_15_ with Differentiation Media vs. Coll_15_ with Standard Media | No | ns | 0.1681 |
| Coll_15_ with Differentiation Media vs. Coll_15_-PyKC_1_ with Differentiation Media | Yes | * | 0.0329 |
| Coll_15_ with Differentiation Media vs. Coll_15_-PyKC_1_ with Standard Media | No | ns | 0.9100 |
| Coll_15_ with Differentiation Media vs. Coll_15_-PyKC_2_ with Differentiation Media | Yes | * | 0.0286 |
| Coll_15_ with Differentiation Media vs. Coll_15_-PyKC_2_ with Standard Media | No | ns | 0.9995 |
| Coll_15_ with Differentiation Media vs. TCP with Differentiation Media | No | ns | 0.9923 |
| Coll_15_ with Differentiation Media vs. TCP with Standard Media | No | ns | 0.1115 |
| Coll_15_ with Differentiation Media vs. TCP with Differentiation Media + LPS | No | ns | >0.9999 |
| Coll_15_ with Standard Media vs. Coll_15_-PyKC_1_ with Differentiation Media | Yes | **** | <0.0001 |
| Coll_15_ with Standard Media vs. Coll_15_-PyKC_1_ with Standard Media | Yes | ** | 0.0084 |
| Coll_15_ with Standard Media vs. Coll_15_-PyKC_2_ with Differentiation Media | Yes | **** | <0.0001 |
| Coll_15_ with Standard Media vs. Coll_15_-PyKC_2_ with Standard Media | Yes | * | 0.0495 |
| Coll_15_ with Standard Media vs. TCP with Differentiation Media | Yes | * | 0.0255 |
| Coll_15_ with Standard Media vs. TCP with Standard Media | No | ns | >0.9999 |
| Coll_15_ with Standard Media vs. TCP with Differentiation Media + LPS | No | ns | 0.1161 |
| Coll_15_-PyKC_1_ with Differentiation Media vs. Coll_15_-PyKC_1_ with Standard Media | No | ns | 0.4279 |
| Coll_15_-PyKC_1_ with Differentiation Media vs. Coll_15_-PyKC_2_ with Differentiation Media | No | ns | >0.9999 |
| Coll_15_-PyKC_1_ with Differentiation Media vs. Coll_15_-PyKC_2_ with Standard Media | No | ns | 0.1184 |
| Coll_15_-PyKC_1_ with Differentiation Media vs. TCP with Differentiation Media | No | ns | 0.2058 |
| Coll_15_-PyKC_1_ with Differentiation Media vs. TCP with Standard Media | Yes | **** | <0.0001 |
| Coll_15_-PyKC_1_ with Differentiation Media vs. TCP with Differentiation Media + LPS | No | ns | 0.0506 |
| Coll_15_-PyKC_1_ with Standard Media vs. Coll_15_-PyKC_2_ with Differentiation Media | No | ns | 0.3935 |
| Coll_15_-PyKC_1_ with Standard Media vs. Coll_15_-PyKC_2_ with Standard Media | No | ns | 0.9974 |
| Coll_15_-PyKC_1_ with Standard Media vs. TCP with Differentiation Media | No | ns | >0.9999 |
| Coll_15_-PyKC_1_ with Standard Media vs. TCP with Standard Media | Yes | ** | 0.0049 |
| Coll_15_-PyKC_1_ with Standard Media vs. TCP with Differentiation Media + LPS | No | ns | 0.9603 |
| Coll_15_-PyKC_2_ with Differentiation Media vs. Coll_15_-PyKC_2_ with Standard Media | No | ns | 0.1049 |
| Coll_15_-PyKC_2_ with Differentiation Media vs. TCP with Differentiation Media | No | ns | 0.1845 |
| Coll_15_-PyKC_2_ with Differentiation Media vs. TCP with Standard Media | Yes | **** | <0.0001 |
| Coll_15_-PyKC_2_ with Differentiation Media vs. TCP with Differentiation Media + LPS | Yes | * | 0.0443 |
| Coll_15_-PyKC_2_ with Standard Media vs. TCP with Differentiation Media | No | ns | >0.9999 |
| Coll_15_-PyKC_2_ with Standard Media vs. TCP with Standard Media | Yes | * | 0.0307 |
| Coll_15_-PyKC_2_ with Standard Media vs. TCP with Differentiation Media + LPS | No | ns | >0.9999 |
| TCP with Differentiation Media vs. TCP with Standard Media | Yes | * | 0.0154 |
| TCP with Differentiation Media vs. TCP with Differentiation Media + LPS | No | ns | 0.9985 |
| TCP with Standard Media vs. TCP with Differentiation Media + LPS | No | ns | 0.0750 |

**Reference:**

1 Koh, L. B. *et al.* Epoxy cross-linked collagen and collagen-laminin Peptide hydrogels as corneal substitutes. *J Funct Biomater* **4**, 162-177, doi:10.3390/jfb4030162 (2013).
